# Complete Chloroplast Genome of *Paphiopedilum delenatii* and Phylogenetic Relationships among Orchidaceae

**DOI:** 10.3390/plants9010061

**Published:** 2020-01-02

**Authors:** Huyen-Trang Vu, Ngan Tran, Thanh-Diem Nguyen, Quoc-Luan Vu, My-Huyen Bui, Minh-Tri Le, Ly Le

**Affiliations:** 1 Faculty of Biotechnology, Nguyen Tat Thanh University, District 4, Hochiminh City 72820, Vietnam; vthtrang@ntt.edu.vn (H.-T.V.); nthanhdiem1708@gmail.com (T.-D.N.); huyen99xx@gmail.com (M.-H.B.); 2 Faculty of Biotechnology, International University-Vietnam National University, Linh Trung Ward, Thu Duc District, Hochiminh City 7000000, Vietnam; kimngan.tran@hdr.qut.edu.au (N.T.); lequangminhtri@gmail.com (M.-T.L.); 3 Tay Nguyen Institute for Scientific Research, Vietnam Academy of Science and Technology, Dalat 670000, Vietnam; vuquocluan07@yahoo.com.vn; 4 Vingroup Big Data Institute, Hai Ba Trung District, Hanoi 100000, Vietnam

**Keywords:** *Paphiopedilum*, chloroplast genome, comparative genomics, conservation genomics

## Abstract

*Paphiopedilum delenatii* is a native orchid of Vietnam with highly attractive floral traits. Unfortunately, it is now listed as a critically endangered species with a few hundred individuals remaining in nature. In this study, we performed next-generation sequencing of *P. delenatii* and assembled its complete chloroplast genome. The whole chloroplast genome of *P. delenatii* was 160,955 bp in size, 35.6% of which was GC content, and exhibited typical quadripartite structure of plastid genomes with four distinct regions, including the large and small single-copy regions and a pair of inverted repeat regions. There were, in total, 130 genes annotated in the genome: 77 coding genes, 39 tRNA genes, 8 rRNA genes, and 6 pseudogenes. The loss of *ndh* genes and variation in inverted repeat (IR) boundaries as well as data of simple sequence repeats (SSRs) and divergent hotspots provided useful information for identification applications and phylogenetic studies of *Paphiopedilum* species. Whole chloroplast genomes could be used as an effective super barcode for species identification or for developing other identification markers, which subsequently serves the conservation of *Paphiopedilum* species.

## 1. Introduction

The sequence of chloroplast (cp) genomes can provide information for studying genetic relationships, gene transfer, cloning, and domestication of species [1]. Much research has shown the role of plastomes in practice. The project of chloroplast genome sequencing and barcoding *Fritillaria* [2] helped identify *Fritillaria* species for medical use. The sequencing of the chloroplast genome of the rice variety Nagina-22 [3] enriched genetic resources to support the breeding and crossbreeding of next-generation rice varieties. Na Tian et al. (2018) sequenced and analyzed the chloroplast genome of *Epipremum aureum* that contributed to the propagation and support of gene transfer of this medicinal plant [4]. Shuai Guo et al. (2018) successfully sequenced the chloroplast genome of *Paeonia ostii* which enhanced the productivity of this medicinal herb [5]. In orchids, the sequencing and analysis of cp genomes helped explain phylogenetic relationships and the evolutionary path of Orchidaceae [6,7].

Currently in Vietnam, only *Panax vietnamesis* (Ngoc Linh Ginseng), a valuable endemic Vietnamese ginseng, has had the chloroplast genome sequenced based on four species (two of *Panax vietnamensis*, one of *P. bipinnatifidus*, and one of *P. stipuleanatus*). From then, the study analyzed phylogenetic species and identified four potential indicators for molecular barcode for classification of this target group [8].

*Paphiopedilum* are the favorite ornamental plants in the world including in Vietnam. Due to its natural and specific beauty, *Paphiopedilum* is hunted and traded in large numbers, leading to danger of extinction. Vietnam is the country with the largest number of *Paphiopedilum* species in the world. However, according to IUCN (International Union for Conservation of Nature) statistics, of nearly 24 species of Vietnamese *Paphiopedilum*, 23 species are on the list of threatened with extinction [9]. *Paphiopedilum delenatii* (Cypripedioideae, Orchidaceae) is usually found at the elevations of 300–750 m and is distributed mostly in the southern region of Vietnam. *P. delenatii* has important ornamental value and is being subjected to overexploitation and habitat destruction. According to the IUCN, *P. delenatii* is now a critically endangered (CR) species with approximately 200 mature individuals left. Controlling over-exploitation and illegal trade is difficult, in which official protectors need to have basic knowledge of identification techniques to distinguish valuable and common species. *Paphiopedilum* species are easily recognized by their specific flower morphology. However, most illegal trades are with immature, non-flowered plants, which leads to species misidentification. The situation of uncontrolled exploitation and smuggling subsequently leads to the destruction of more *Paphiopedilum* species. Timely identification and control helps to limit illegal collection and smuggling, reducing the risk of extinction.

Identifying species using molecular techniques is considered to be the most effective because they give high accuracy results and help to quickly and accurately identify species using a very small number of samples from plant parts, such as roots, stems, leaves. Because of this, an understanding of genomics plays a crucial role. Divergent and conserved regions in the genome provide useful information to establish DNA-based [10,11,12,13,14] as well as PCR-based [13,15,16,17,18,19,20] identification markers, supporting the protection and management of species. However, in Orchidaceae, most of the studies are of the Epidendroideae subfamily. There were up to 99 complete chloroplast genomes of Epidendroideae species in the RefSeq database (accessed on 8 August 2019). In contrast, there are 3 of Vanilloideae, 3 of Apostasioideae, 9 of Orchidoideae, and 7 of Cypripedioideae. Up to now, 4 cp genomes of *Paphiopedilum* (belong to Cypripedioideae) have been sequenced and analyzed, i.e., *P. tranlienianum* [21], *P. dianthum* [22], *P. armeniacum*, and *P. niveum* [23]. Hence, this study contributes to data resource of chloroplast genomes of Cypripedioideae, in particular, and of Orchidaceae, in general.

**Figure 1:**
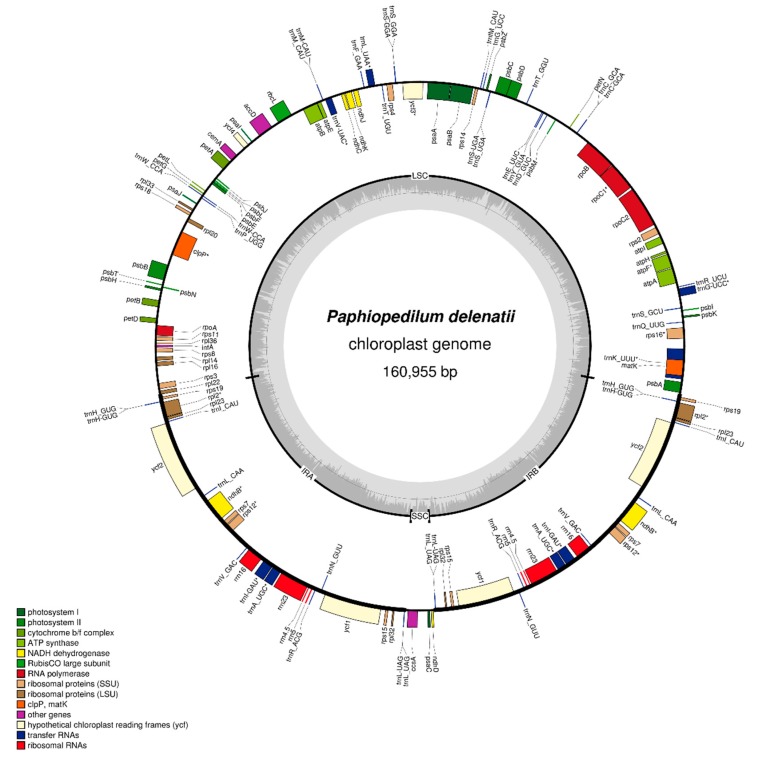
Gene map of the *Paphiopedilum delenatii* chloroplast genome. Annotated genes are colored according to functional categories. Genes lying outside of the outer circle were transcribed clockwise, while those inside the circle were transcribed counterclockwise. The innermost, darker gray corresponds to GC content, while the lighter gray corresponds to AT content. IR, inverted repeat; LSC, large single-copy region; SSC, small single-copy region; SSU, small subunit; LSU, large subunit; RNA, ribonucleic acid; NADH, nicotinamide adenine dinucleotide (NAD) + hydrogen (H); ATP, Adenosine Triphosphate; RubisCO, Ribulose-1,5-bisphosphate carboxylase/oxygenase.

For the above reasons, we carried out next-generation sequencing of *P. delenatii* (Figure 1), an endemic species of Vietnam, and assembled its complete chloroplast genome. Our end goal was to extend the genetic resources for the endangered *P. delenatii* and *Paphiopedilum* species, in general.

## 2. Results and Discussion

### 2.1. Chloroplast Genome of Paphiopedilum delenatii

The whole genome of *P. delenatii* was sequenced from total DNA and resulted in 11.6 million high-quality paired-end reads with high read coverage of 800×. The complete chloroplast genome was then assembled separately following the procedure of our previous study [24]. To verify the plastid genome sequence, we independently compared it to four barcoded markers, i.e., *mat*K, *trn*L-UAA, *rpo*B, and *rpo*C1 (GenBank accessions MK792631, MK787353, MK876160, and MK792704, respectively), belonging to the chloroplast genome of the same *P. delenatii* sample that was sequenced by the Sanger method in our previous study [25]. The pairwise-alignment results, which showed 100% similarity between single regions of the assembled genome, proved that the whole plastid genome sequence was of *P. delenatii*. This complete plastome sequence was deposited in GenBank under accession MK463585.

The assembled chloroplast genome is 160,955 base pairs (bp) in length (Figure 1). It exhibits a typical quadripartite structure of the large single-copy (LSC, 89,869 bp) and small single-copy (SSC, 2694 bp) regions, separated by a pair of inverted repeat regions (IRs, 34,196 bp each). There are 107 unique genes that were annotated (Table 1), including 68 protein-coding genes, 30 transfer RNA genes, 4 ribosomal RNA genes, and 5 pseudogenes. In particular, there are 23 genes in double copies (Table 1).

The overall GC content of *P. delenatii* is 35.6%. The GC contents of the LSC, SSC, and IR regions are 33.0%, 28.5%, and 39.3%, respectively (Table 2). The GC content in the IR region was higher than both LSC and SSC regions in all examined *Paphiopedilum* plastomes. This result agreed with previous studies [6,7,26,27]. The presence of four ribosomal RNA (rRNA) genes is considered to be the reason for high GC contents in the IR regions [6,26]. However, another hypothesis proposed that the higher GC content evolution in IR regions does not relate to natural selection but to GC-biased gene conversion (gBGC) [7,27]. Accordingly, GC to AT mutations were unstable while AT to GC ones were fixed after gBGC, and hence the gBGC process prefers repairing DNA mismatches in recombining DNA over evolutionary time [28]. Meanwhile, IRs are considered to be recombination hotspots due to their identical inverted repeat structures, which increase the frequency of intraplastomic recombination [29,30] and hence increase gBGC.

GC content has been reported to be different not only between different regions of a genome, but also between different genomes of different species [31,32]. Overall GC content of some orchid plastomes were recorded, e.g., *Dendrobium moniliforme* (37.54%), *Goodyera schlechtendaliana* (37.07%), *Vanilla aphylla* (35.02%) [33], *Cremastra appendiculata* (37.2%), *Calanthe davidii* (36.9%), *Epipactis mairei* (37.2%), and *Platanthera japonica* (37%) [6], with their plastome lengths ranging from 148,778 to 162,835 bp. GC content of the *Paphiopedilum* species in our study range from 35% to 35.6%, with genome lengths of 154.699 to 162.682 bp, similar to other orchids. Hence, *Paphiopedilum* and *Vanilla* contain lower a GC percent, which has been suggested to mean that the sequence variability was higher toward the enrichment of AT nucleotides [33,34]. The difference in GC content of the nuclear genome has been proposed as a useful value for identification of species [31]. Karimi et al. (2018) introduced the GCSpeciesSorter tool for accurately and quickly determining GC content and, hence, classifying species in a mix of DNA relationships for metagenomic studies [35]. Hence more studies on the GC content of plant plastomes might provide a useful measurement for the identification of species.

The comparison of four *Paphiopedilum* plastomes showed that *P. delenatii* inherits a similar conserved plastome structure to its *Paphiopedilum* sisters (Table 2). Some differences are that *P. niveum* has the least total gene number and coding sequence (CDS) number and has no protein-coding gene *inf*A. *P. dianthum* contains four genes, *ycf*68(× 2) and *orf*42(× 2), that are not observed in the other three plastomes. *P. delenatii* is distinguished by double copies of *trn*L_UAG and *rpl*32 (Table S1).

In *P. delenatii*, there are six *ndh* genes, all of which are pseudogenes: *ndhB*(× 2)*, ndhC, ndhD, ndhJ,* and *ndhK*. *ndhA, ndhE, ndhF, ndhG, ndhH,* and *ndhI* are entirely absent from the *P. delenatii* cp genomes. The same applies to *P. armeniacum* and *P. niveum* chloroplast genomes. As for *P. dianthum*, the RefSeq annotation showed two pseudogenes: *ndhB* and *ndhD*. This result was consistent with the report of Guo et al. (2012) [36]. However, we aligned the *ndhC*, *ndhJ*, and *ndhK* sequences of *P. delenatii* to the three chloroplast genomes of *Paphiopedilum* and found a version of *ndhJ* present in the *P. dianthum* cp genome (Figure S1).

**Figure 2:**
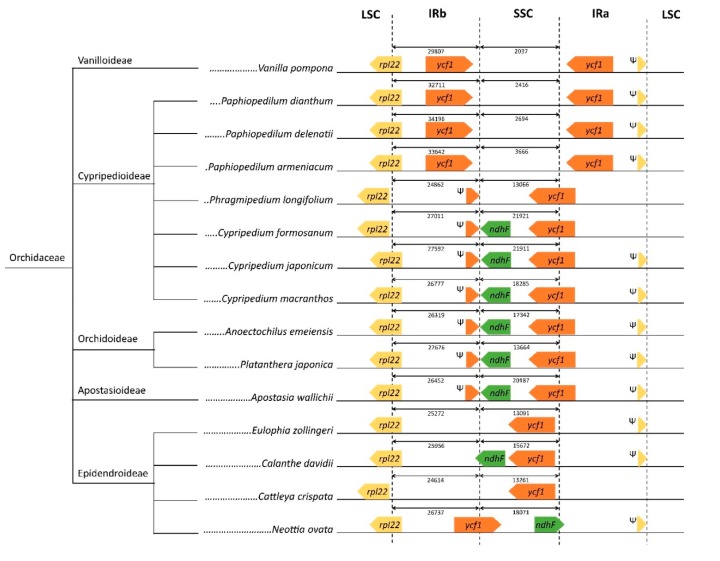
Schematic representation of the borders between inverted repeats (IRs) and large single-copy (LSC) and small single-copy (SSC) of *P. delenatii* and other 15 species from all five subfamilies of Orchidaceae. Ψ indicates a pseudogene.

*Ndh* genes code for the enzyme NADH dehydrogenase [37,38] that is responsible for electron transport of chloroplasts. The loss and variation of *ndh* genes in IR boundaries in orchid species have been the focus of multiple investigations [23,39,40]. Here, we compared the boundaries between two inverted repeat regions, i.e IRa and IRb, and LSC and SSC regions of *P. delenatii* to those of 14 other orchid species from 5 subfamilies of Orchidaceae (Figure 2). All Cypripedioideae species with high-resolution plastid genome annotations were included. *P. delenatii* exhibited a highly-conserved pattern of IR boundaries to those of *P. dianthum*, *P. armeniacum*, as well as *Vanilla poompona* (Vanilloideae): *rpl22* at the IRb/LSC border, 2 copies of the *ycf1* gene in IRs, and a pseudogenized *rpl22* next to the border of IRa/LSC. These 4 species possessed rather small SSCs (2037–3666 bp) compared to the remaining 11 species (13,066–21,921 bp). Interestingly, in the other four Cypripedioideae species, which were *Phragmipedium longifolium*, *Cypripedium formosanum*, *Cypripedium japonicum*, and *Cypripedium macranthos*, *ycf1* genes shifted to the borders of IRs and SSC, leaving one functional *ycf1* at the SSC/IRa junction and one pseudogene in IRb, next to the IRb/SSC. The three *Cypripedium* species had the *ndhF* gene in the SSC region, which was absent in all *Paphiopedilum*. These Cypripedioideae had very similar features in IR junctions to those of Orchidoideae and Apostasioideae. In Epidendroideae, IR boundaries of the 4 examined species exhibited higher heterogeneity: *ndhF* is present in *Calanthe davidii* and *Neottia ovata*, but not in *Eulophia zollingerri* and *Cattleya crispate*; *ycf1* is mostly in the SSC region but at the IRb/SSC border in *Neottia ovata*. In the case of *P. niveum*, the IR regions, which contain six palindromic pairs instead of one (Table S2), were not determined. Hence, this species was not included in Figure 2 and its plastome structure should be interpreted further.

The IR region is highly conserved and stable in the chloroplast genome. The expansion and contraction of the IR region is a common characteristic of the chloroplast genome. Luo et al. (2014) proposed four types of IR/SSC junction when they examined seven orchids [41]. In our study, the IR pattern of *Cypripedium* and Orchidoideae and Apostasioideae species matched with type I; *Phragmipedium longifolium* matched with type III; *Eulophia zollingery* and *Cattleya crispata* matched with type IV. None of our examined species matched with Type II, which was the overlap of Ψ*ycf*1 and *ndh*F genes [41]. Instead, *Vanilla pompona*, *Calanthe davidii*, and *Neottia ovata* expressed entirely new IR patterns (Figure 2). Together with the shift of the *ycf*1 gene, the presence or absence of the *ndhF* gene was one of the factors observed in different IR/SSC patterns. According to Guo et al. [36], among the Cypripedioideae, *Cypripedium* species inherited the *ndh*F gene from their ancestors; this gene has been lost from the other genera. *ndhF* gene loss was previously proposed to be correlated with the instability of IR/SSC boundary [40]. More studies are required to better understand IR evolution. However, this study emphasized the diversity of IR/SSC boundaries in orchids.

### 2.2. Repeat and Microsatellite Analysis

For *Paphiopedilum delenatii*, we found 645 repeats with lengths from 30 to 58 bp (Table S3). The number of forward repeats were the most common (176/645), followed by palindromic repeats (168/645), reverse repeats (167/645), and complement repeats (133/645). Most repeats were inside intergenic spacers. Notably, 23 repeat sequences were entirely located in the *ycf2* gene. There were 87 simple sequence repeats (SSRs) or microsatellite sequences identified in the *P. delenatii* chloroplast genome (Table S4). Fifty SSR loci were in intergenic regions, while 21 were in the coding areas (12 of which were in *ycf1* gene). Most SSR loci were mononucleotide repeats with AT motifs (57/87).

Repeats are units of DNA that are similar in the genome. There are short repeats and longer repeats. SSR (simple sequence repeat), or microsatellite, is a type of low-complexity, short repeat with 1–6 nucleotides. Generally, microsatellite SSRs are widely distributed throughout the genome and have a great effect on recombination and rearrangement of the genome [42,43]. In our study, there were no tetranucleotide or longer repeats in the cp genome. This result was consistent with previous reports that most SSRs include mono- and di-nucleotide repeats while tri-, tetra-, penta-, and hexa-nucleotide repeat sequences were detected at much lower frequencies [6]. The longer repeat, known as a minisatellite, contains 10–100 nucleotides. In term of direction and complementary, these repeats are divided into four types: forward (direct) repeats, reverse repeats (also known as inverted repeat –IR), complement repeats, and palindromic repeats (reverse complement repeat) [44]. Both microsatellite and minisatellite repetitive sequences play significant roles in species identification. SSRs were used as DNA barcodes to clear identify 5 genotypes of *Solanum melongena* L. by Chinnappareddy et al. [45]. In Orchidaceae, SSR markers were developed and utilized as identification tools in various studies [18,46,47,48] due to their high reproducibility and variability [45,49,50]. In particular, SSRs in the chloroplast genome were reported to have a high level of polymorphism among species and loci [51]. Sets of cp SSRs were also isolated and developed for recognizing valuable plants, serving conservation genetics, investigation of chloroplast genetic structure, adaptive evolution, and population [52,53,54,55,56,57]. Even single loci, i.e., *trn*L and *trn*L-F, in chloroplast were used to develop specific primers for amplifying SSR sequences for a population genetic study [58]. The longer repeat minisatellites were supposed to have specific mechanisms of evolution and function, forming common, as well as unique, repeat patterns [44,59], hence they are important tools for taxonomic and phylogenetic studies. Eight out of 13 species in the *Phoenix* genus (Arecaceae) were unambiguously distinguished using minisatellites developed from a 700 bp region in the chloroplast spacer *trn*G (GCC)-*trn*fM (CAU) [60]. Our study provided primary data of SSRs and minisatellite repeats for further research on identifying applications.

### 2.3. Phylogenetic and Species Resolution Analyses

**Figure 3:**
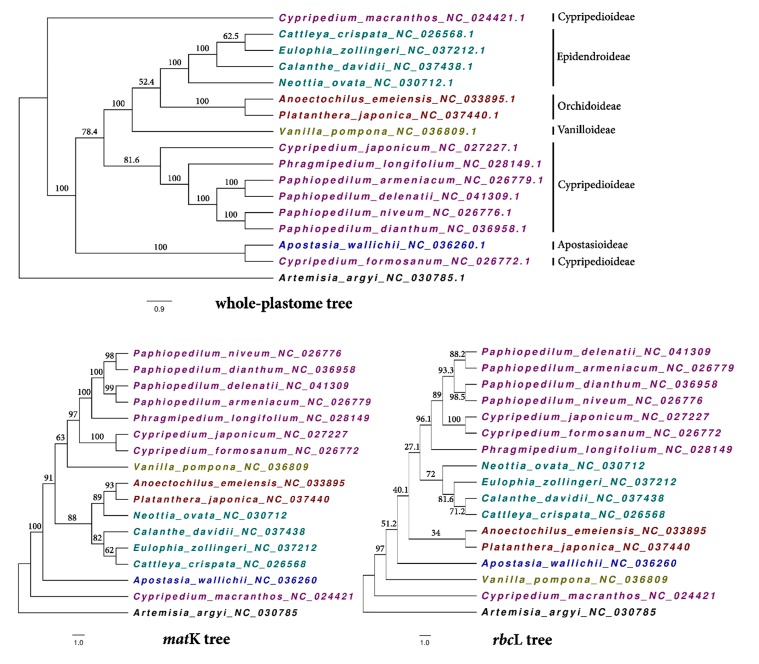
Cladogram of the phylogenetic relationship between P. delenatii and other Orchidaceae species based on maximum likelihood analysis. The number on the branches are bootstrap values.

We included chloroplast genomes of *P. niveum* and all the Orchidaceae species examined in the above IR boundaries section to understand the phylogenetic relationship between *P. delenatii* and other species across subfamilies (Figure 3). *Artemisia argyi* was set as the outgroup. Firstly, a whole-plastome tree was constructed. As expected, *P. delenatii* along with other *Paphiopedilum* again showed a high similarity and close phylogenetic relationship to each other. The 6 species of Cypripedioideae were clustered together into one clade, excluding *C. macranthos* and *C. formosanum*. Although the IR boundary analysis showed similar patterns between *V. pompona* and those of *Paphiopedilum* accessions, in term of the whole plastome, *V. pompona* was more similar to Epidendroideae and Orchidoideae. Next, we constructed phylogenetic trees based on two popular short barcodes: *mat*K and *rbc*L. All three phylogenetic trees presented a full separation of 16 species. This might be because the analysis consisted of distinct species. In contrast to the plastome tree, *Apostasia wallichii* was now segregated in an independent monophyletic branch, as expected, since it belongs to a different subfamily. Furthermore, *C. formosanum* was grouped with the members of its genus. A dot plot similar analysis on the whole-genome alignment of *P. delenatii* to other species (Figure S2) found that *C. formosanum* and *A. wallichii* both had an inverse similarity fragments located in the LSC region, at the position of 10,000–80,000 bp. This led to greater genetic similarities between *C. formosanum* and *A. wallichii*, which grouped them into one clade in the whole-plastome tree. *C. macranthos* was closer to its sisters of Cyprideoideae in terms of nucleotide polymorphism (Figures S3 and S4), but differed in terms of amino acid variations, which explains the separation of this species in the phylogenetic trees.

The chloroplast genome was proposed to be used as meta-barcode by some previous studies [61,62,63,64]. Hence, two universal mini-barcodes, *mat*K and *rbc*L, were established in order to compare the effects of species resolution under the tree-based method. From the above analyses, using the whole genome for phylogenetic relationship analysis might not be practical due to the unexpected problem of inversion. However, for barcoding, the species separation, but not relationship, is of first concern. In this respect, using the whole plastome as a super-barcode is effective. Besides, we also analyzed genetic distance matrices among species using plastome, *mat*K and *rbc*L, data independently (Table S5). The average, minimum, and maximum of distances were calculated. Except for *rbc*L with a low average value (0.034), the average genetic distance by *mat*K (0.122) and by plastome (0.112) were all high. Although the average values were not much different between plastomic and *mat*K data, the minimum value of *matK* (0.005) was much lower than that of the plastome (0.012). While some previous studies showed that *matK* was not able to identify all species in some close taxonomic groups [65,66], our result suggests that the entire cp genome could do better than *mat*K in identifying closely related species.

### 2.4. Divergence of Hotspot Regions

**Figure 4:**
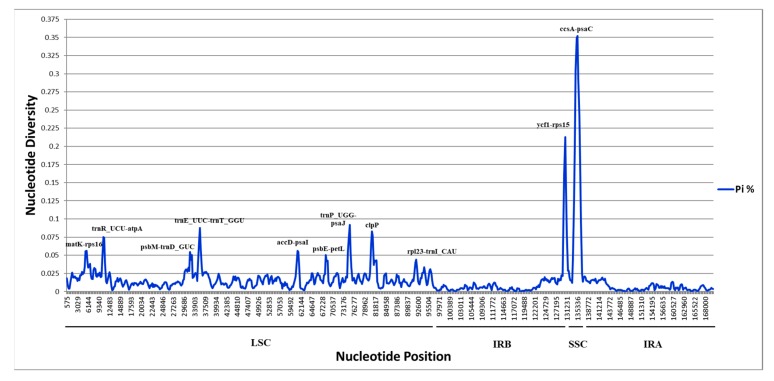
Sliding window of nucleotide diversity from the alignment of four *Paphiopedilum* plastomes. (Pi %) Parsimony rate; (IR) inverted repeat regions; (LSC) large single-copy region; (SSC) small single-copy region; red line: diversity threshold.

We calculated the nucleotide variability in the chloroplast genomes of the four analyzed species: *Paphiopedilum delenatii*, *Paphiopedilum armeniacum*, *Paphiopedilum niveum*, and *Paphiopedilum dianthum*. The *P. delenatii* plastome was highly conserved compared to other cp genomes of *Paphiopedilum* species. The number of single nucleotide polymorphism (SNP) was 24,211 out of 170,423 bp of the alignment. The values of nucleotide diversity (pi) ranged from 0 to 0.34889 and the diversity threshold was 0.079. However, the diversity threshold decreased at 0.0377 when the SSC regions were excluded from the analysis. At this threshold, 11 highly-variable regions were suggested as potential markers in species identification and phylogeny study of the *Paphiopedilum* genus (Figure 4). These 11 highly-variable regions included 1 protein-coding gene (*clpP*) and 8 intergenic spacers (*mat*K*-rps*16, *trn*R_UCU*-atp*A, *psb*M*-trn*D_GUC, *trn*E_UUC*-trn*T_GGU, *acc*D*-psa*I*, psb*E*-pet*L, *trn*P_UGG*-psa*J, and *rpl*23*-trn*L_CAU) in the LSC region; 1 intergenic spacer (*ycf*1*-rps*15) in the repeat region IRb; and 1 intergenic spacer (*ccs*A*-psa*C) in the SSC region (Table S6).

In the study of Bi et al. (2018), nucleotide diversity of eight *Fritillaria* species ranged from 0 to 0.02583 [67]. The pi value was from 0 to 0.05872 for the nine cp genomes of *Eragrostis* species in the study of Somaratne et al. (2019) [68]. In comparison with those studies, the pi value (0–0.34889) in our study was much higher. The reason was that there were two fragments with extremely high divergence within the SSC region that were not present in the mentioned studies. Therefore, we tried another analysis in which the SSC region was removed. The threshold was significantly decreased, at 0.0377 instead of 0.079, for the whole cp genomes. The high divergence of SSC was also reported recently by Cui et al. (2019). A comparison of 10 ginger species showed an average value of nucleotide variability of 0.0187, while it was only 0.0075 when comparing 4 species of the same family [69]. Therefore, SSC seemed to rapidly evolve compared to LSC. The two inverted regions, IRa and IRb, were quite conserved, with low nucleotide diversity. In addition, the narrow endemism is also a prominent feature of *Paphiopedilum* species. Of the known species, 72% are narrowly endemic with very limited distribution [70]. The local distribution and ecological separation might be the reason for considerable genetic differences between species in the same genus and might clarify the high nucleotide diversity of *Paphiopedilum* species in our study.

Using short sequences for the identification of a species is still the current universal method due to its simple and time-saving traits. Indeed, the average divergence of *mat*K (0.122) was even higher than that of the plastome (0.112) in our study. The reason for this is that the plastome contains both diverse and conserved regions while *mat*K is a selected, high-variable locus [71]. This result proved the species separation capability of this mini-barcode in comparison with the super one. Common markers, such as the internal transcribed spacer (ITS) in the nucleus and cp loci *rbc*L, *mat*K, *ycf*1, *trn*L, *trn*L-F, *atp*F-*atp*H, *trn*H-*psb*A, etc., were the most used. However, as no single locus can resolve the entire plant species [72,73], new variable sites are still being sought [6,10,12,74,75]. In the chloroplast genome, SNPs were not random but clustered as “hotspots” [76], which were defined as highly-variable loci [77]. A series of new findings for species-specific barcodes was developed recently. Protein-coding gene *clpP* and intergenic spacer *rps*15-*ycf*1 from our proposal were also recommended previously [76,78,79,80]. The hot region of *trn*E-*trn*T was matched with the study of Zhao et al. (2018) [77]. Although *rps*16 and its intergenic spacer with *trn*Q were suggested in much research [10,11,26,74,81], our study introduced a new intergenic spacer of this gene, i.e., *rps*16-*mat*K. However, searching for new variable sites as candidate barcodes is just the first step. Primer designing and amplification success are also required for barcoding effects [10,25,74].

Besides barcoding applications, hot region information is also used for developing other PCR-based identification techniques. rDNA-ITS (internal transcribed spacer) sequences were used to design species-specific SCAR (sequence characterized amplified regions) markers, in which 3 primer pairs—SCAR-600armF/Pap-ITS2R, SCAR-300delF/Pap-ITS2R, and SCAR-700micF/Pap-ITS2R—were effectively used to amplify and recognize three *Paphiopedilum* species (*P. armeniacum*, *P. micranthum*, and *P. delenatii*) and their hybrids [17]. Similarly, two DNA sequences, *rpo*C2 and *atp*F-*atp*H, were reported to contain species-specific SNPs, insertions, and deletions. This information was utilized to develop 3 species-specific primers for quickly identifying species: *Cypripedium guttatum* var. *koreanum*, *C. japonicum*, and *C. formosanum* of the Korean *Cypripedium* genus. This ARMS (amplification refractory mutation system) method was also based on electrophoresis technique without a sequencing step [15]. In another study (2014), the divergent nucleotide sequence of the ITS region and 3 cpDNA fragments were amplified and subsequently cut with several restriction enzymes to create species-specific types of DNA patterns. The PCR-restriction fragment length polymorphism (PCR-RFLP) approach was successful in the identification of 25 native *Dendrobium* species in Thailand [16].

## 3. Materials and Methods

### 3.1. Plant Material, DNA Extraction, and Sequencing

**Figure 5:**
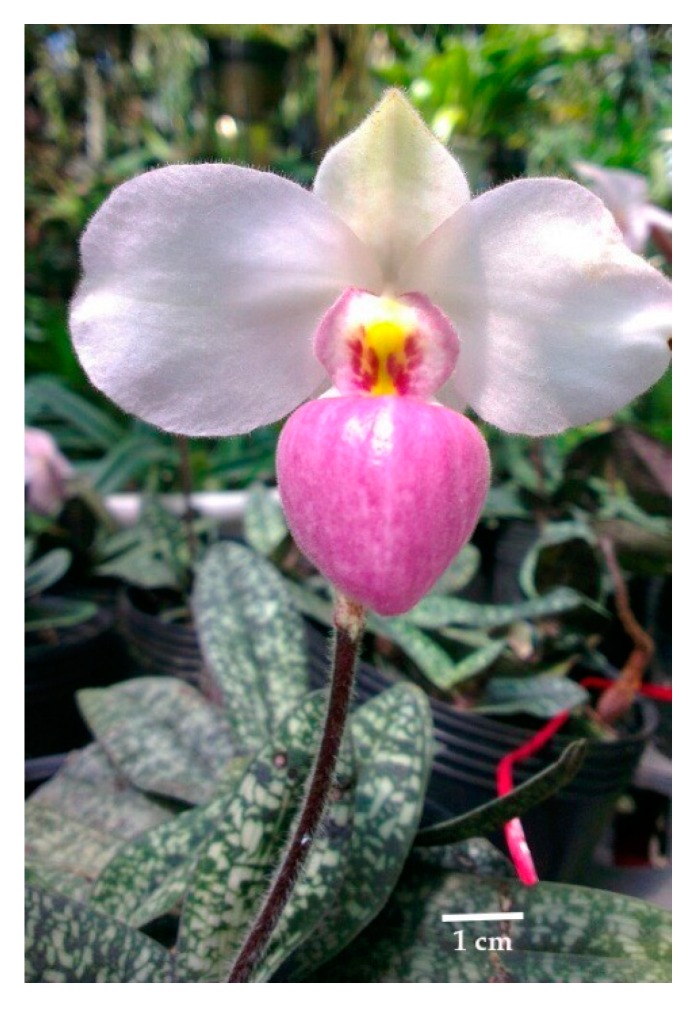
The flower of endemic species *Paphiopedilum delenatii.*

The sample of *Paphiopedilum delenatii* plant (Figure 5) was identified by the shape of the flowering plant and stored at −80 °C at Tay Nguyen Biological Institute, Vietnam. First, 0.2 g of leaf was ground with 5 µL proteinase K, 3mL of a mixture of beta-mer and extract buffer at 65 °C, then incubated for 30 min at 65 °C. The sample was had 600 uL P:C:I added and was centrifuged for 10 min at 10,000 rpm. After adding 5 uL of RNAse and incubating at 37 °C, the sample had 600 uL C:I added. DNA was precipitated by isopropanol and incubated overnight at −20 °C. The pellet, obtained by centrifugation, was washed with 70%, 80%, and 90% ethanol. DNA was suspended in 25 uL TE and stored at −20 °C. The library construction and whole-genome sequencing of *P. delenatii* was performed by GENEWIZ (South Plainfield, NJ, USA). Sequencing was carried out on an Illumina HiSeq (Genewiz, South Plainfield, USA) using a 2 × 150 paired-end (PE) configuration.

### 3.2. Read Data Processing and Chloroplast Genome Assembly

Demultiplexing was performed by bcl2fastq 2.17. Raw data was filtered as follows: (1) discard pair-end reads with adapter, (2) discard pair-end reads when the content of N bases is more than 10% in either read, and (3) discard pair-end reads when the ration of bases of low quality (Q < 20) is more than 0.5 in either read. The chloroplast genome of *P. delenatii* was reconstructed using NOVOPlasty 2.7.2 [82], with the complete chloroplast genome of *P. armeniacum* (RefSeq: NC_026779.1) as the reference genome and the *rbcL* from the same plastid genome of *P. armeniacum* as the seed sequence. The annotation was done by GeSeq [83] and further manually curated by comparison to the annotations of *P. armeniacum*, *P. dianthum*, and *P. niveum* in GenBank. The genome map was drawn by OGDRAW [84].

### 3.3. Repeat Sequence and Microsatellite Identification

REPuter [85] was used to calculate DNA repeats, including forward, reverse, complement, and palindromic kinds of repeat sequences. The repeats were identified with a hamming distance of 3 and minimum repeat size of 30 [6]. MISA [86] was used to identify microsatellite sequences with default parameters.

### 3.4. Examination of IR Junctions

We manually examined the IR junctions of all included orchid species. Annotations of IRs, SSC, LSC, and genes were based on their respective annotations in the RefSeq database. For genomes without IR annotations, we used REPuter to identify their pairs of inverted repeats.

### 3.5. Phylogenetic Analysis

The phylogenetic analysis was based on the complete genome sequences of 16 orchid species under the maximum likelihood criterion and the GTR + I + G nucleotide substitution model using R package phangorn [87]. Node was calculated from 1000 bootstrap replicates. Figtree [88] was used to visualize the resulting tree. The multiple alignment data of 16 plastomes was used to calculate variable sites and genetic distance matrices using MEGA [89]. MAFFT [90] was used to pairwise align and construct dot-plot graph of these 16 plastomes.

### 3.6. Nucleotide Variability Calculation

DnaSP v6.1 was used to extract the parsimony variable site density over the plastid genome alignment of four analyzed *Paphiopedilum* species with a sliding window (window length ≤ 600 and step size = 200). Nucleotide diversity was calculated by the ratio of Pi and window length. The diversity threshold was 0.079, calculated by the sum of the average and double the standard deviation [67]. Regions with diversity higher than the threshold were recommended as highly variable regions.

## 4. Conclusions

In this study, we aimed to expand the genetic resource of the endangered species *P. delenatii* by next-generation sequencing and chloroplast genome assembly. *P*. *delenatii* chloroplast genome exhibited a quadripartite structure of 160,955 bp and a total of 130 genes, which were highly conserved compared to other *Paphiopedilum* species. All six *ndh* genes in the *P. delenatii* cp genome were pseudogenes. The presence or absence of the *ndh*F gene and the shift of *ycf*1 and *rpl*22 genes on the boundaries between IRs and LSC and SSC regions resulted in different IR/SSC patterns that can be useful in inferring species relationships. A reference of 87 SSRs/minisatellite repeats in *P. delenatii* was proposed for further research on identification applications. Eight highly-variable regions were suggested as the potential markers in barcoding and phylogeny studies of *Paphiopedilum* genus. Hence, the sequence data of *P. delenatii* complete chloroplast genome could be used directly in the identification of *Paphiopedilum* species or for the development of other identification markers, such as SSR, barcoding, or species-specific PCR-based techniques. Although it is still costly and time-consuming compared to short DNA sequencing, genome sequencing costs have decreased significantly in recent years. Along with the development of other whole genome sequencing without amplification steps, e.g., Nanopore technique, we might hope that sequencing of the whole genome would be easier and convenient in the future.

## Figures and Tables

**Table 1:** List of annotated genes in the *Paphiopedilum delenatii* chloroplast genome.

Classification of Genes		Name of Genes	Number
RNA genes	Ribosomal RNAs	*rrn*4.5(× 2), *rrn*5(× 2), *rrn*16(× 2), *rrn*23(× 2)	8
	Transfer RNAs	*trn*A_UGC(× 2), *trn*C_GCA, *trn*D_GUC, *trn*E_UUC, *trn*F_GAA, *trn*fM_CAU, *trn*G_GCC, *trn*G_UCC, *trn*H_GUG(× 2), *trn*I_CAU(× 2), *trn*I_GAU(× 2), *trn*K_UUU, *trn*L_CAA(× 2), *trn*L_UAA, *trn*L_UAG(× 2), *trn*M_CAU, *trn*N_GUU(× 2), *trn*P_UGG, *trn*Q_UUG, *trn*R_ACG(× 2), *trn*R_UCU, *trn*S_GCU, *trn*S_GGA, *trn*S_UGA, *trn*T_GGU, *trn*T_UGU, *trn*V_GAC(× 2), *trn*V_UAC, *trn*W_CCA, *trn*Y_GUA	39
Protein-coding genes	Photosystem I	*psa*A, *psa*B, *psa*C, *psa*I, *psa*J	5
	Photosystem II	*psb*A, *psb*B, *psb*C, *psb*D, *psb*E, *psb*F, *psb*H, *psb*I, *psb*J, *psb*K, *psb*L, *psb*M, *psb*N, *psb*T, *psb*Z	15
	Cytochrome	*pet*A, *pet*B, *pet*D, *pet*G, *pet*L, *pet*N	6
	ATP synthase	*atp*A, *atp*B, *atp*E, *atp*F, *atp*H, *atp*I	6
	Rubisco	*rbc*L	1
	Ribosomal proteins-small units	*rps*11, *rps*12(× 2), *rps*14, *rps*15(× 2), *rps*16, *rps*18, *rps*19(× 2), *rps*2, *rps*3, *rps*4, *rps*7(× 2), *rps*8	16
	Ribosomal proteins-large units	*rpl*14, *rpl*16, *rpl*2(× 2), *rpl*20, *rpl*22, *rpl*23(× 2), *rpl*32(× 2), *rpl*33, *rpl*36	12
	RNA polymerase	*rpo*A, *rpo*B, *rpo*C1, *rpo*C2	4
	Miscellaneous	*acc*D, *ccs*A, *cem*A, *clpP*, *inf*A, *mat*K	6
	Hypothetical chloroplast reading frames (*ycf*)	*ycf*1(× 2), *ycf*2(× 2), *ycf*3, *ycf*4	6
Pseudogenes	NADH dehydrogenase	*ndh*B(× 2), *ndh*C, *ndh*D, *ndh*J, *ndh*K	6
Total			130

(× 2) refers to genes in double copies.

**Table 2:** Basic features of four *Paphiopedilum* chloroplast genomes.

Species	*Paphiopedilum delenatii*	*Paphiopedilum* *armeniacum*	*Paphiopedilum* *niveum*	*Paphiopedilum* *dianthum*
Total length (bp)	160,955	162,682	159,108	154,699
IR length (bp)	34,196	33,641	31,978	32,711
LSC length (bp)	89,869	91,734	89,958	86,861
SSC length (bp)	2694	3666	5194	2416
Total gene number	130	129	126	130
Coding sequence (CDS) number	77	77	74	79
rRNA number	8	8	8	8
tRNA number	39	38	38	38
Pseudogene number	6	6	6	5
Overall GC content (%)	35.6	35.4	35.0	35.0
GC content of IR (%)	39.3	39.0	40.0	39.0
GC content of LSC (%)	33	32.6	32.0	33.0
GC content of SSC (%)	28.5	31.0	29.0	29.0
GenBank accession	MK463585	NC_026779.1	NC_026776.1	NC_036958.1

## Supplementary Materials

The following are available online at https://www.mdpi.com/2223-7747/9/1/61/s1, Figure S1: Alignment of *ndh*C, *ndh*J, and *ndh*K genes on 4 *Paphiopedilum* using SimpleSynteny; Figure S2: Pairwise dot plot cladograms of *P. delenatii* and other Orchids; Figure S3: Nucleotide polymorphism of Orchidaceae species based on *rbc*L region; Figure S4: Amino acid polymorphism and number of variable sites between *C. macranthos* and other Orchidaceae species using *rbc*L sequence; Table S1: List of annotated genes in Paphiopedilum plastomes; Table S2: Positions of some palindromic repeat pairs in *P. niveum*; Table S3: Full list of DNA repeats in *P. delenatii* cp genome; Table S4: Full list of simple sequence repeats (SSRs) in *P. delenatii* cp genome; Table S5: Genetic distance matrix of analysis data; Table S6. Variable sites from plastomes of 4 *Paphiopedilum* species.

## Abbreviation

IUCN	International Union for Conservation of Nature
Cp	chloroplast
CR	Critically endangered
IR	Inverted repeat region
SSC	Small single-copy region
LSC	Large single-copy region
gBGC	GC-biased gene conversion
SSR	Simple sequence repeat
SNP	Single nucleotide polymorphism
