# Supplementary material for: Complete Chloroplast Genome of Paphiopedilum delenatii and Phylogenetic Relationships among Orchidaceae
Source: Plants (Basel). 2020 Jan 2;9(1):61. doi: 10.3390/plants9010061 (PMC7020410; doi:10.3390/plants9010061)
Supplement: Supplementary file 1 [file plants-09-00061-s001.zip › Supplementary Figure S1.docx]

Complete Chloroplast Genome of *Paphiopedilum delenatii* and Comparative analysis of *Paphipodilum s*pecies

# Supplementary Figures


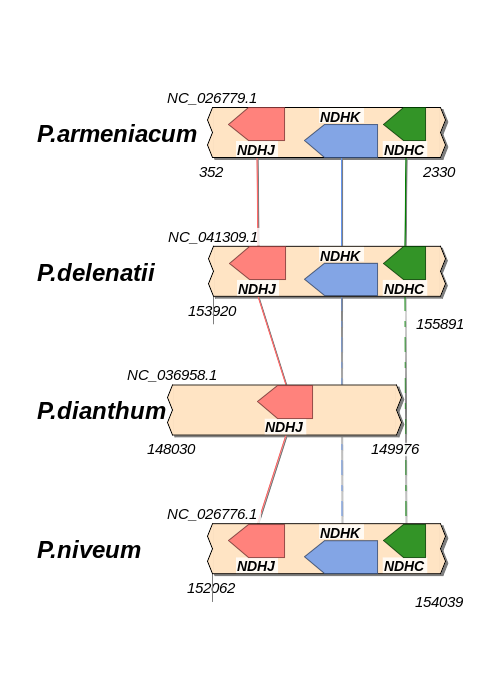


Figure S1. Alignment of *ndhC*, *ndhJ* and *ndhK* genes on 4 *Paphiopedilum* using SimpleSynteny
