# Supplementary material for: Complete Chloroplast Genome of Paphiopedilum delenatii and Phylogenetic Relationships among Orchidaceae
Source: Plants (Basel). 2020 Jan 2;9(1):61. doi: 10.3390/plants9010061 (PMC7020410; doi:10.3390/plants9010061)
Supplement: Supplementary file 1 [file Supplementary Figure S2.pdf › Supplementary Figure S2.pdf]

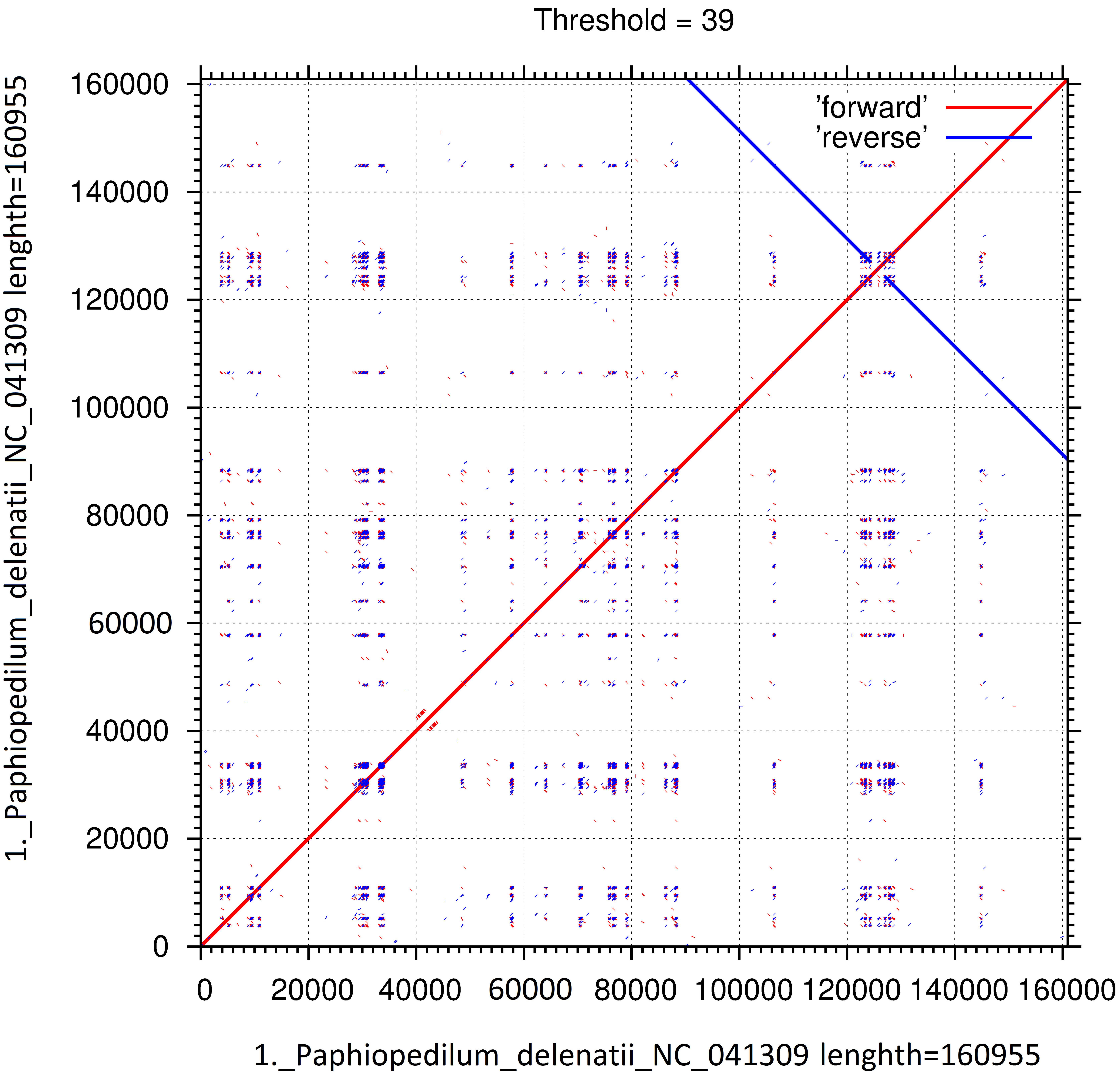
Threshold = 39
'forward'
'reverse'
160000
140000
120000
100000
80000
60000
40000
20000
0
0
20000
40000
60000
80000
100000
120000
140000
160000
1._Paphiopedilum_delenatii_NC_041309 lenghth=160955
1._Paphiopedilum_delenatii_NC_041309 lenghth=160955

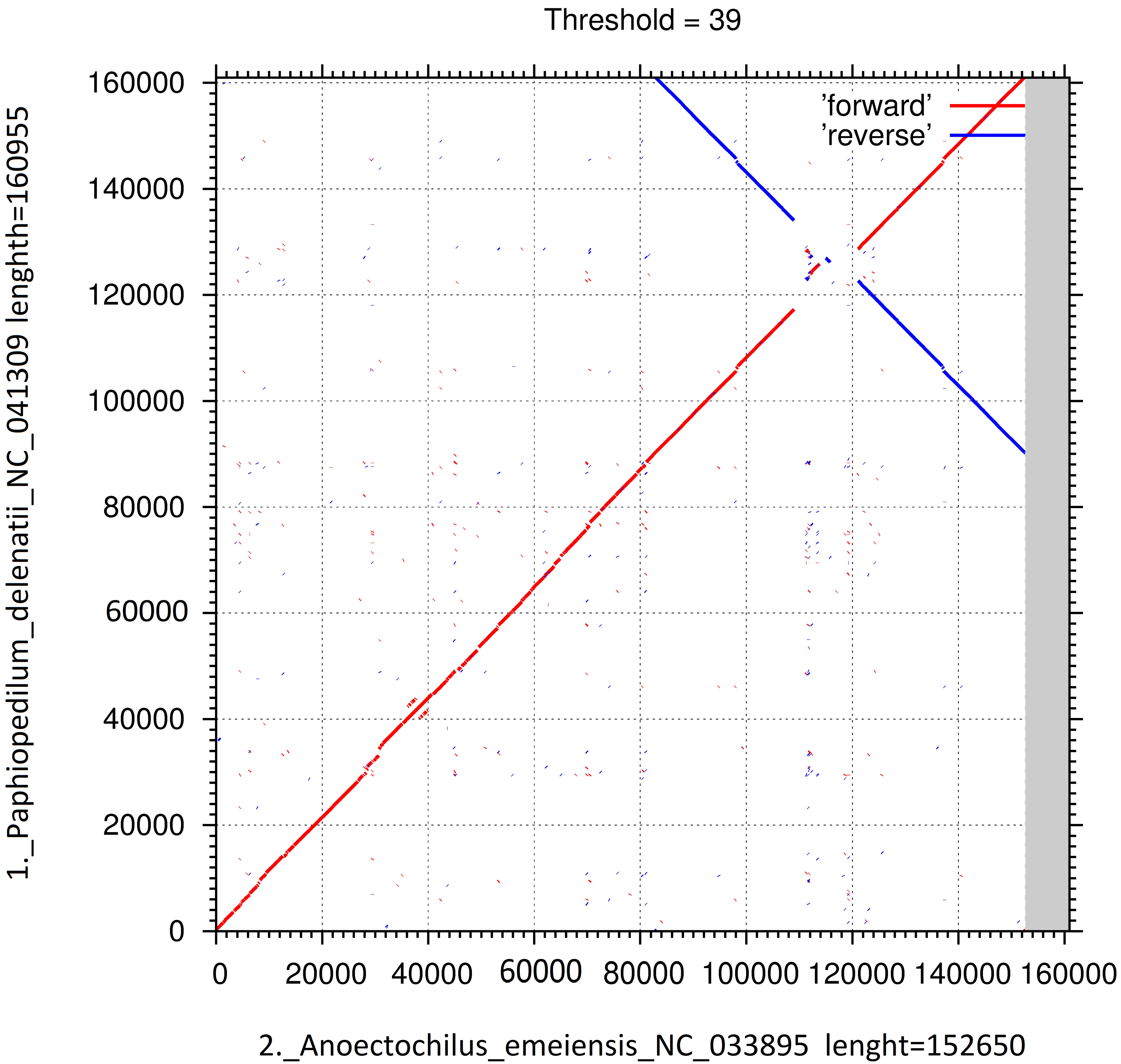

Threshold = 39
'forward'
'reverse'
1._Paphiopedilum_delenatii_NC_041309 lenghth=160955
2._Anoectochilus_emeiensis_NC_033895 lenght=152650
0
20000
40000
60000
80000
100000
120000
140000
160000

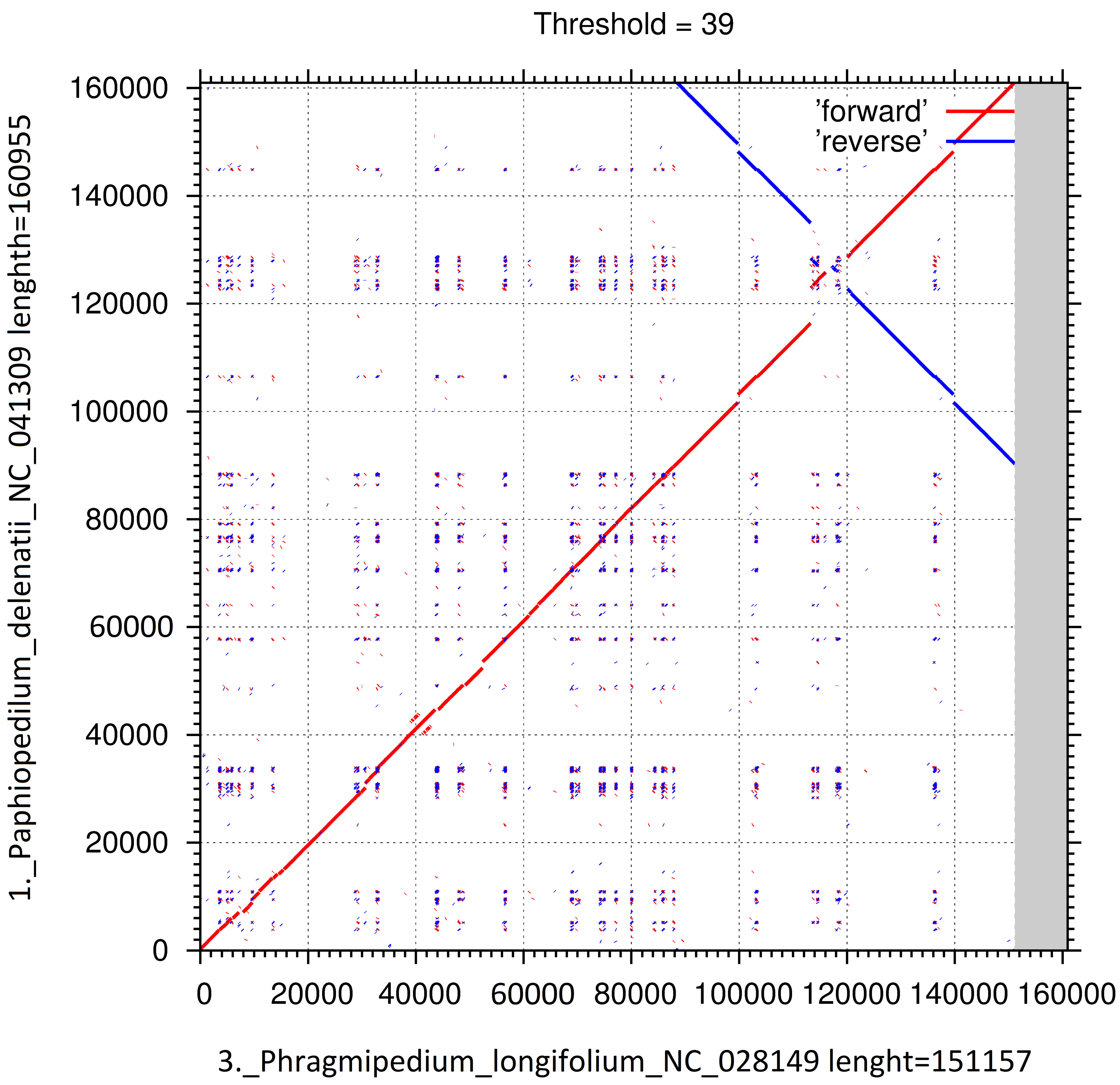

Threshold = 39
'forward'
'reverse'
160000
140000
120000
100000
80000
60000
40000
20000
0
0
20000
40000
60000
80000
100000
120000
140000
160000
1._Paphiopedilum_delenatii_NC_041309 length=160955
3._Phragmipedium_longifolium_NC_028149 lenght=151157

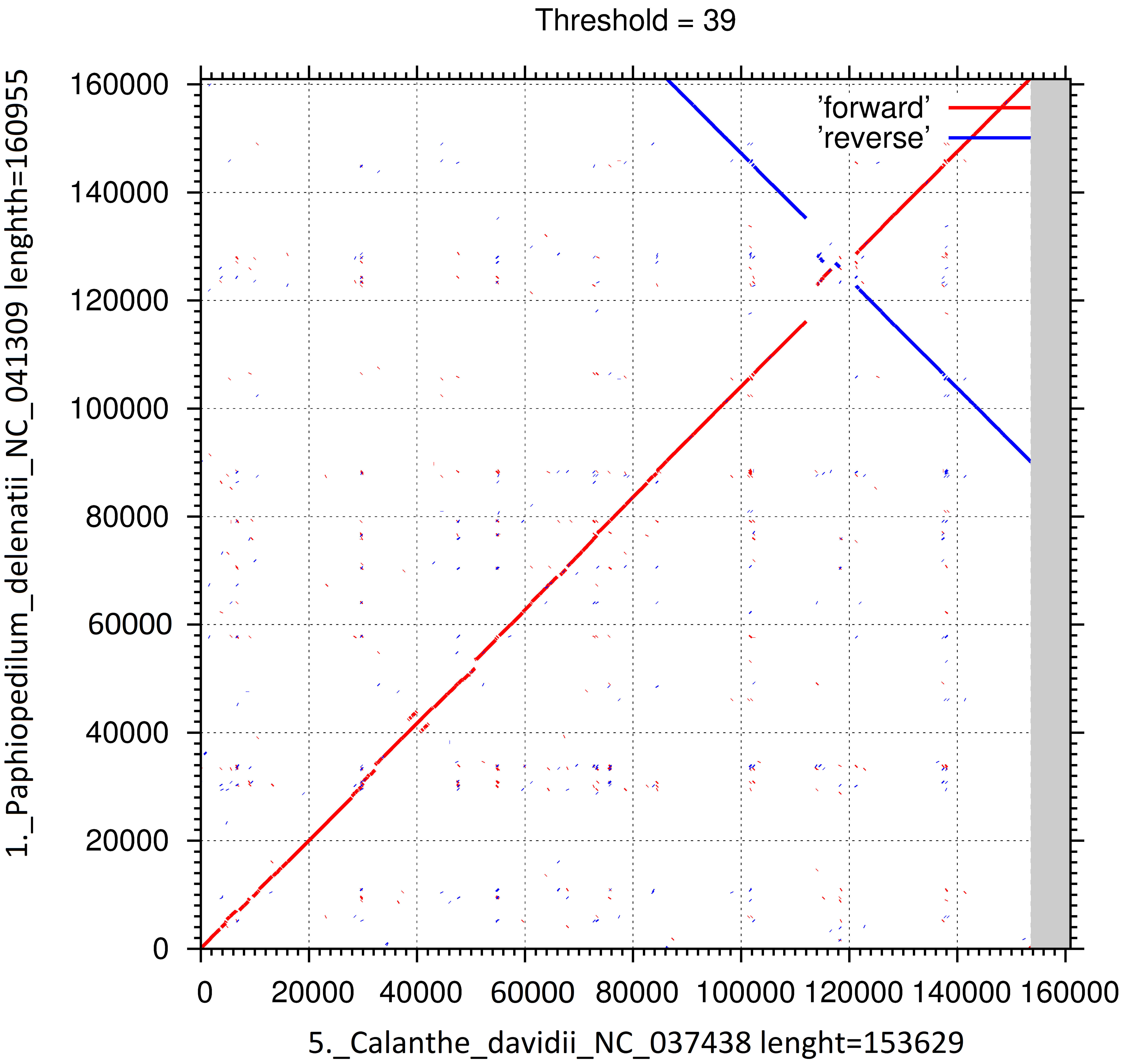

Threshold = 39
'forward'
'reverse'
1._Paphiopedilum_delenatii_NC_041309 lenghth=160955
5._Calanthe_davidii_NC_037438 lenght=153629
0
20000
40000
60000
80000
100000
120000
140000
160000

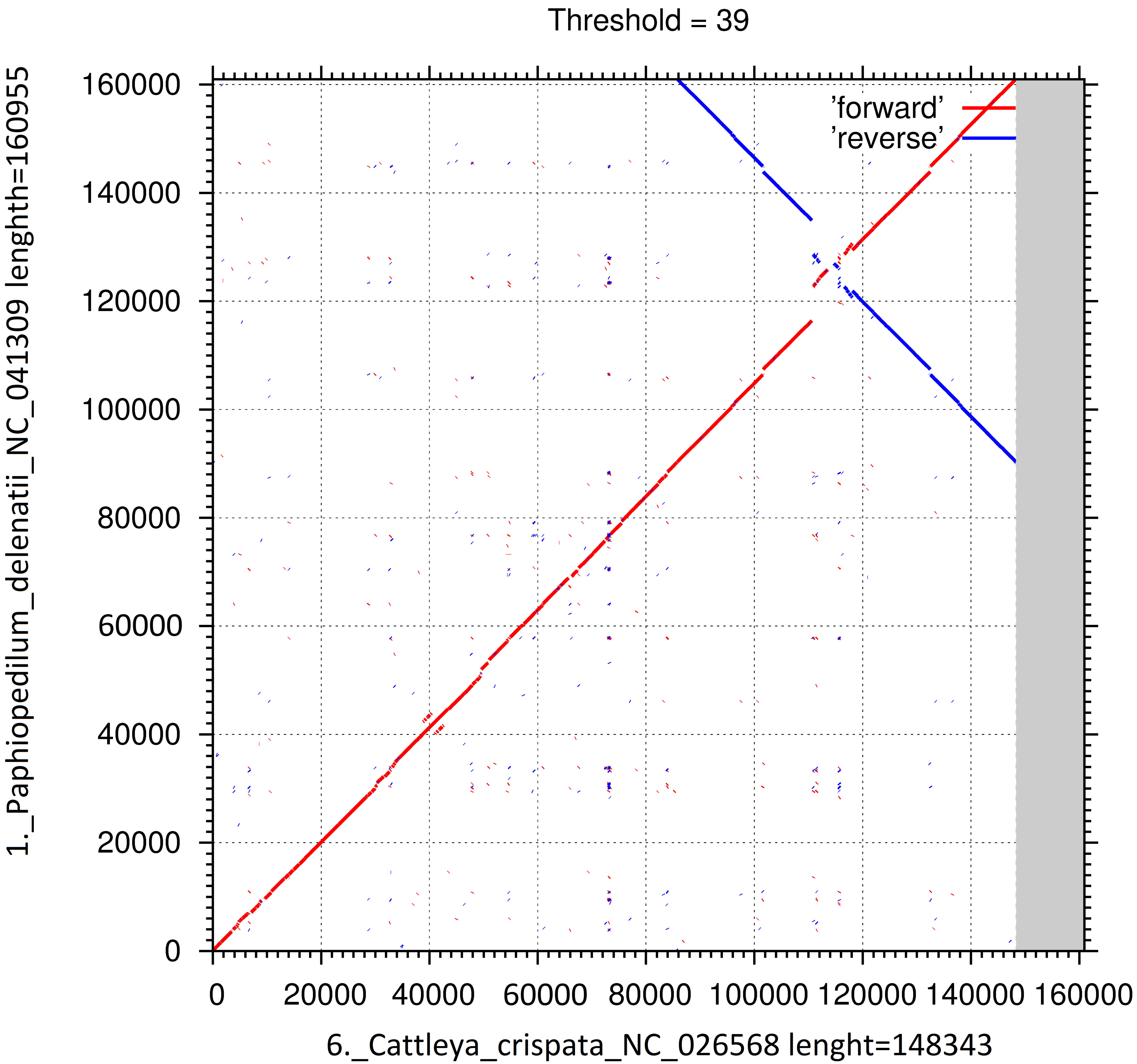

Threshold = 39
'forward'
'reverse'
1._Paphiopedilum_delenatii_NC_041309 lenghth=160955
6._Cattleya_crispata_NC_026568 lenght=148343
0
20000
40000
60000
80000
100000
120000
140000
160000

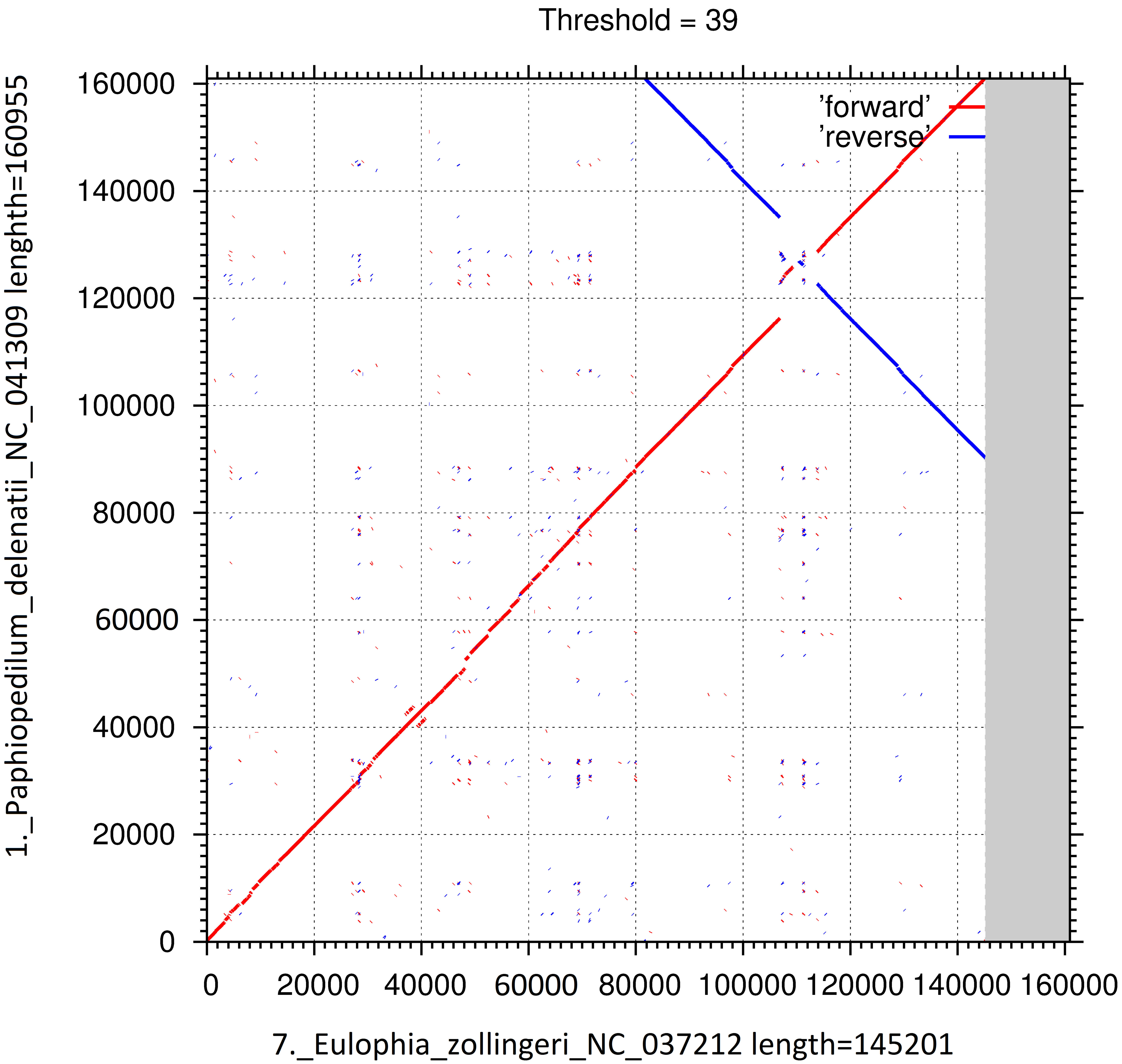

Threshold = 39
'forward'
'reverse'
1._Paphiopedilum_delenatii_NC_041309 lenghth=160955
7._Eulophia_zollingeri_NC_037212 length=145201
0
20000
40000
60000
80000
100000
120000
140000
160000

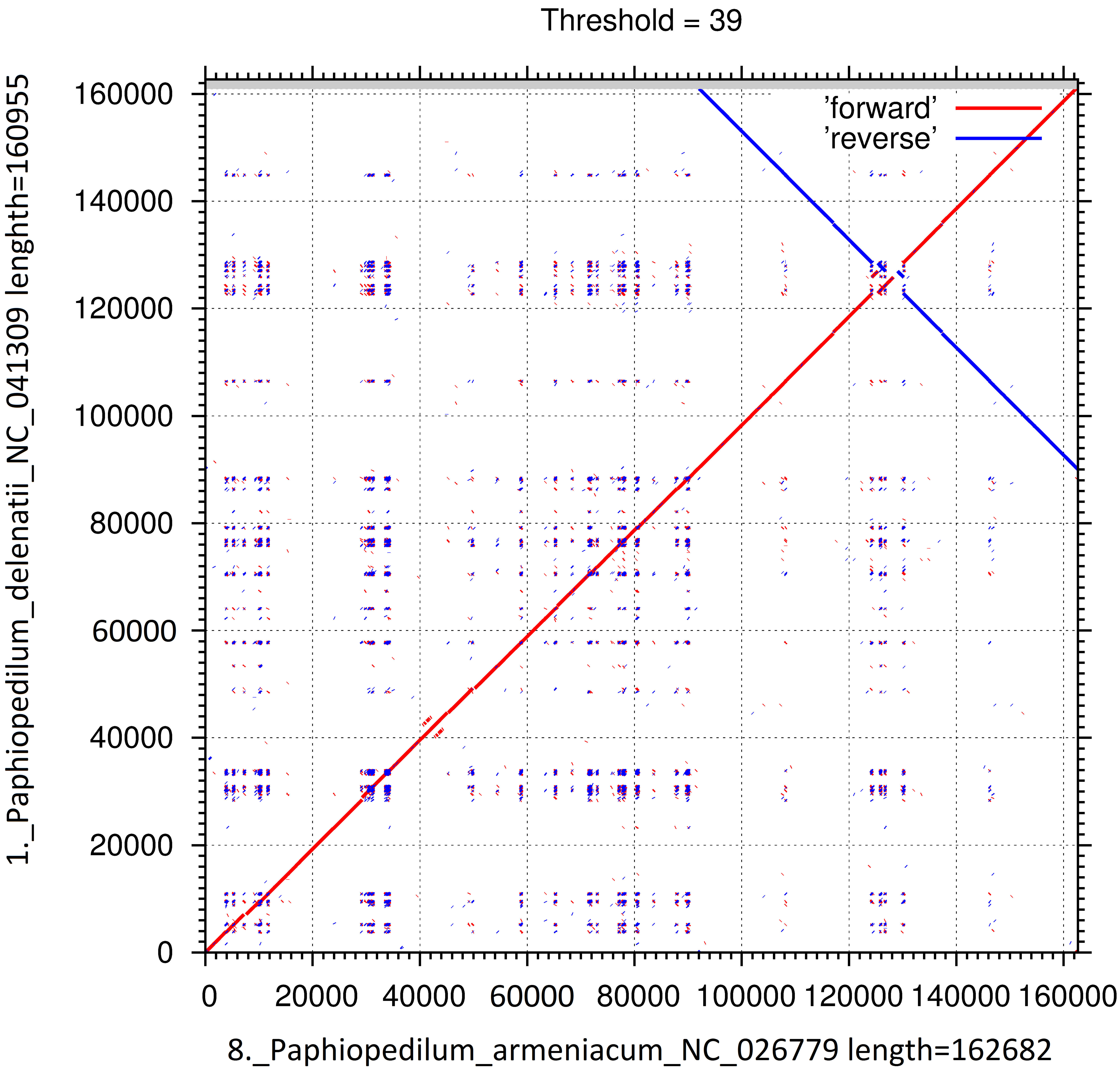

Threshold = 39
'forward'
'reverse'
1._Paphiopedilum_delenatii_NC_041309 lenghth=160955
8._Paphiopedilum_armeniacum_NC_026779 length=162682
0
20000
40000
60000
80000
100000
120000
140000
160000

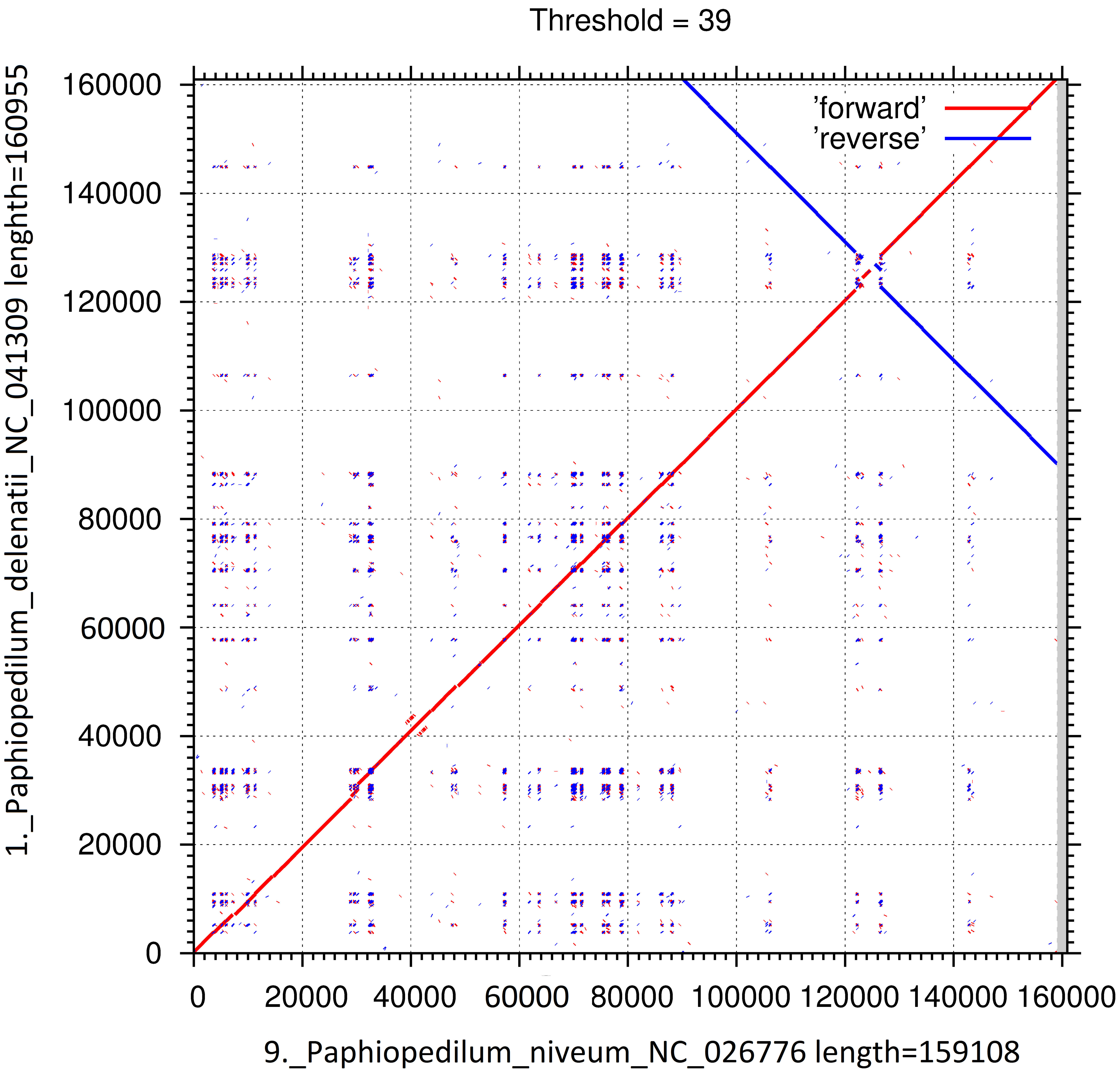

Threshold = 39
'forward'
'reverse'
1._Paphiopedilum_delenatii_NC_041309 length=160955
9._Paphiopedilum_niveum_NC_026776 length=159108
0
20000
40000
60000
80000
100000
120000
140000
160000

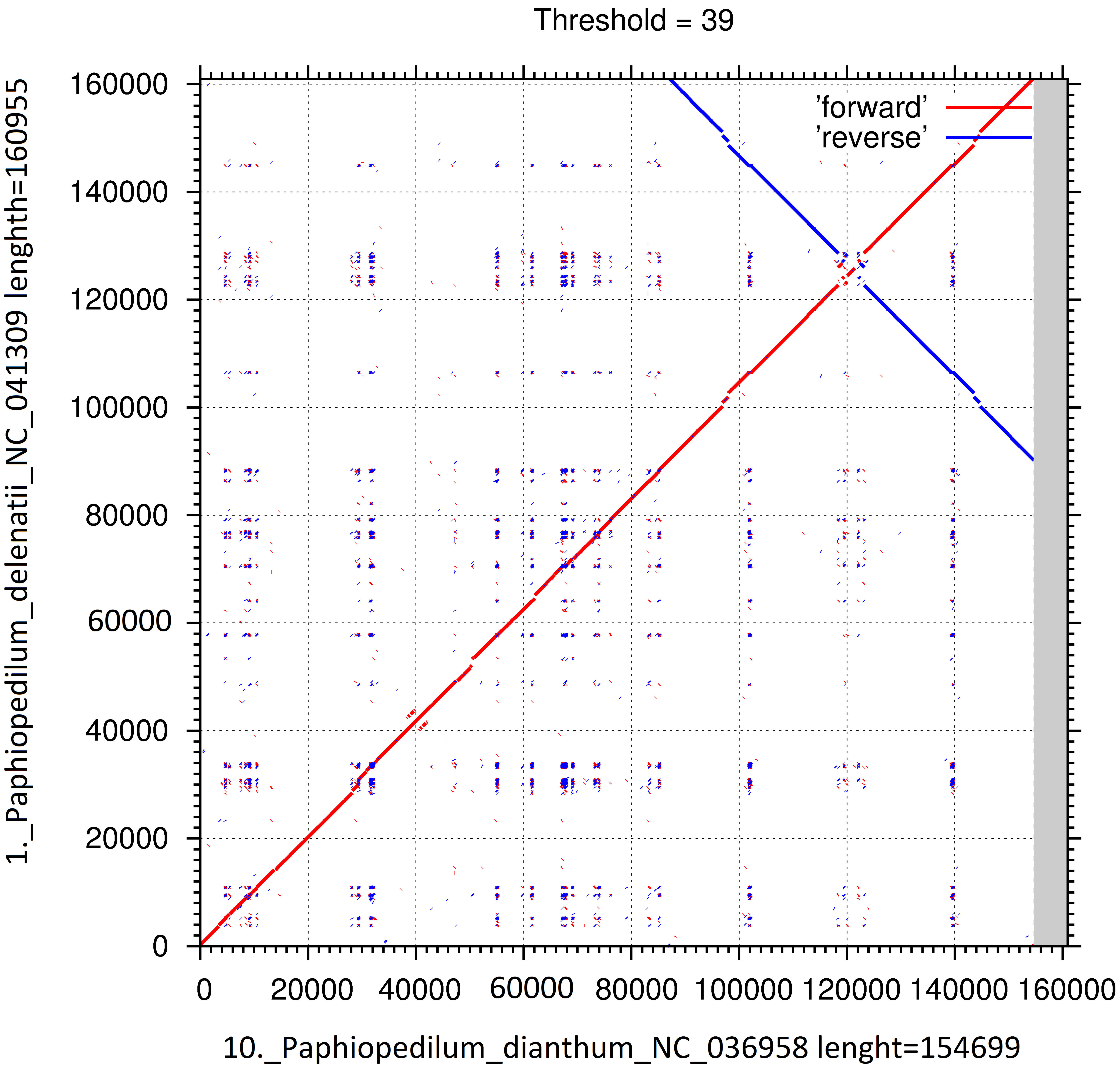

Threshold = 39
'forward'
'reverse'
0
20000
40000
60000
80000
100000
120000
140000
160000
1._Paphiopedilum_delenatii_NC_041309 length=160955
10._Paphiopedilum_dianthum_NC_036958 lenght=154699

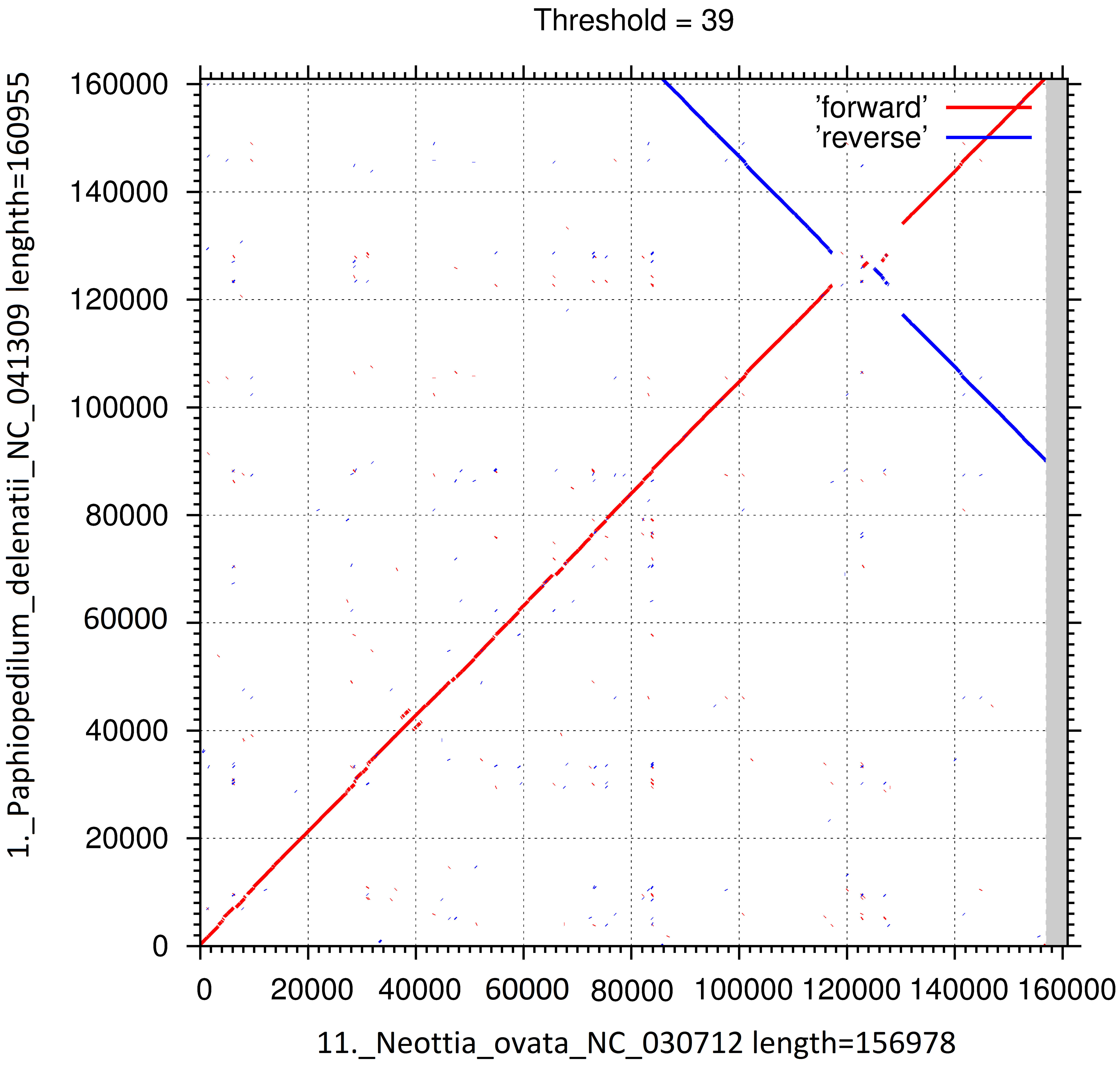

Threshold = 39
'forward'
'reverse'
1._Paphiopedilum_delenatii_NC_041309 lenghth=160955
11._Neottia_ovata_NC_030712 length=156978
0
20000
40000
60000
80000
100000
120000
140000
160000

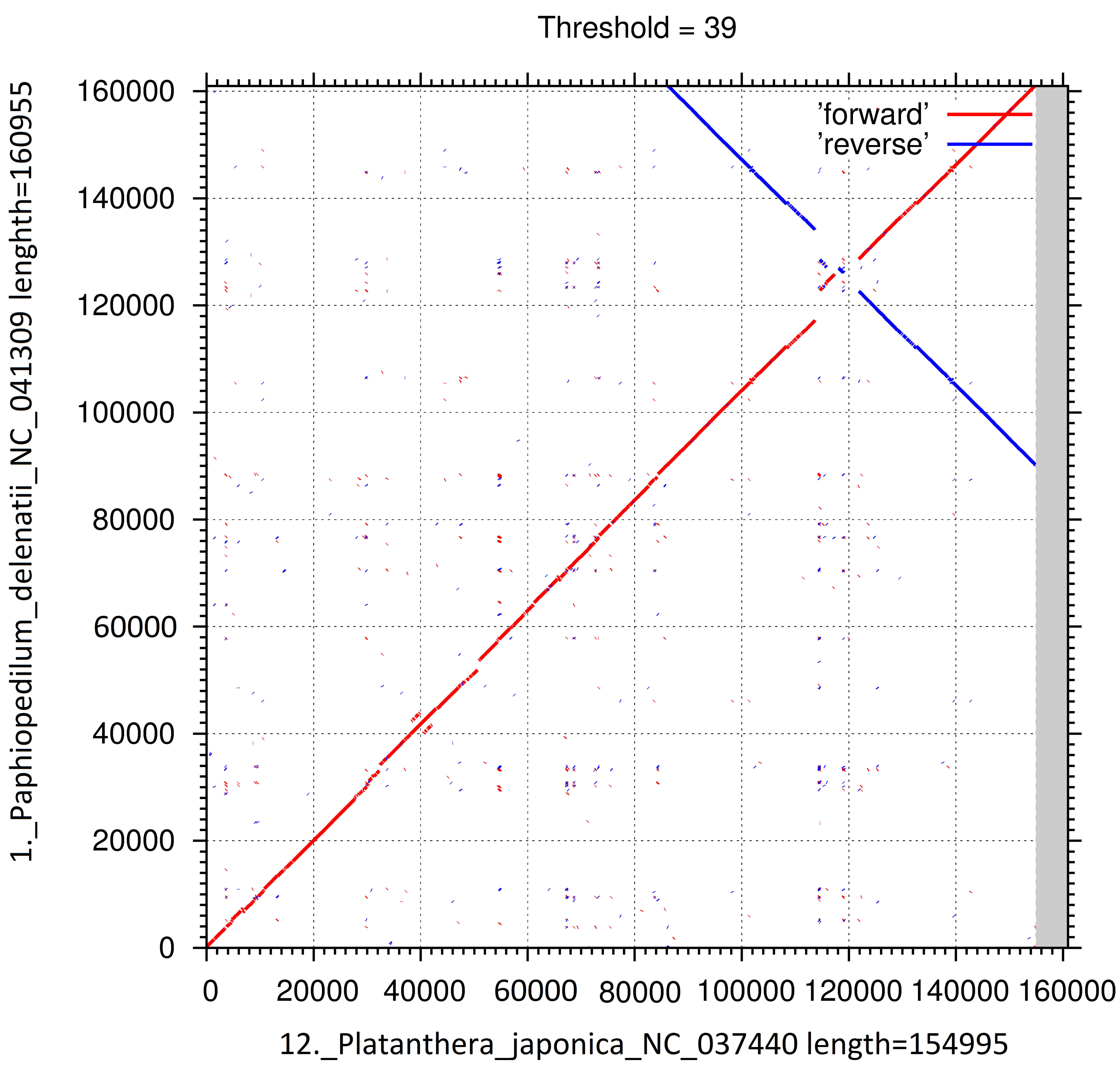

Threshold = 39
'forward'
'reverse'
160000
140000
120000
100000
80000
60000
40000
20000
0
0
20000
40000
60000
80000
100000
120000
140000
160000
1._Paphiopedilum_delenatii_NC_041309 lenghth=160955
12._Platanthera_japonica_NC_037440 length=154995

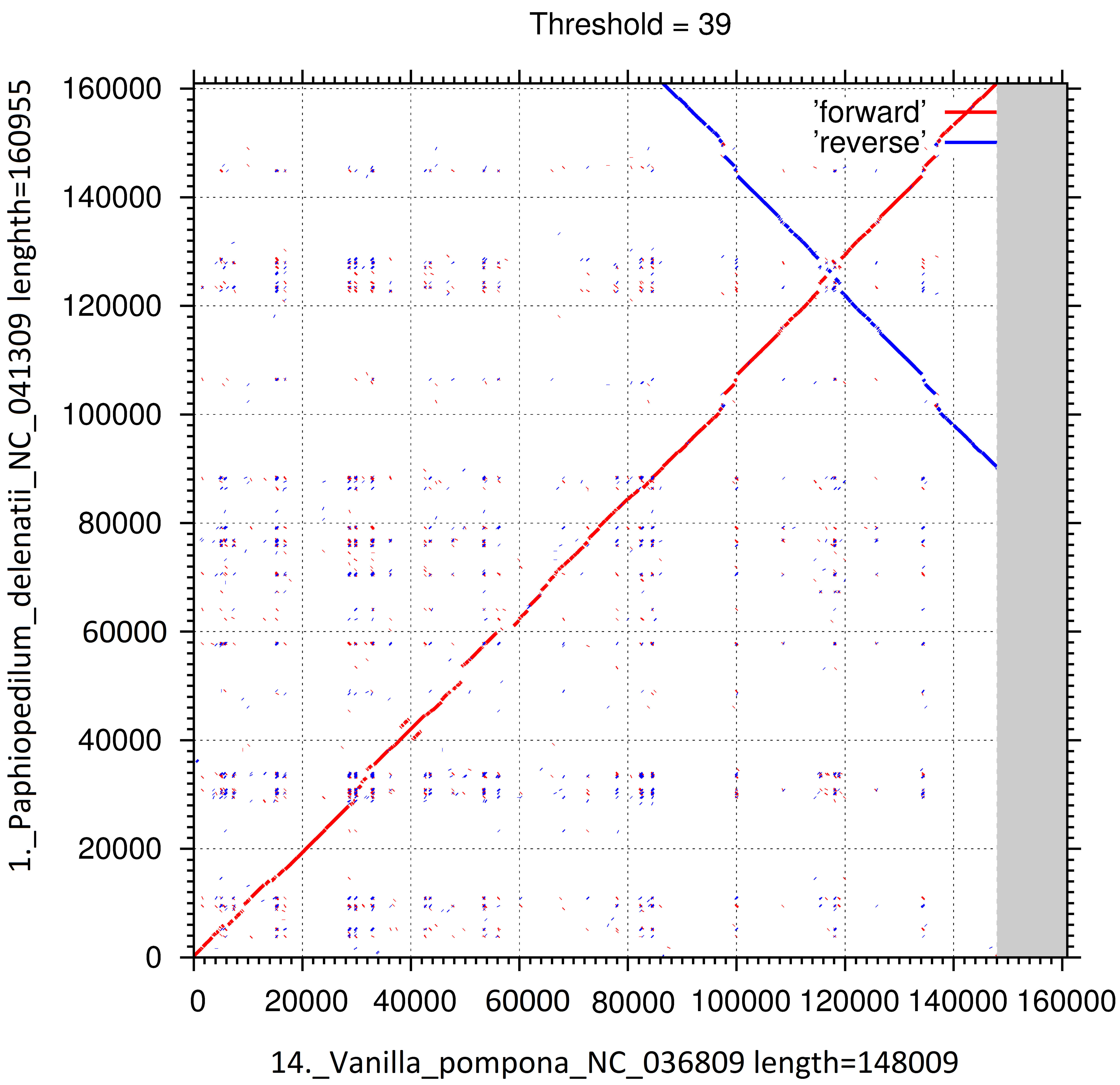

Threshold = 39
'forward'
'reverse'
1._Paphiopedilum_delenatii_NC_041309 lenghth=160955
14._Vanilla_pompona_NC_036809 length=148009
0
20000
40000
60000
80000
100000
120000
140000
160000

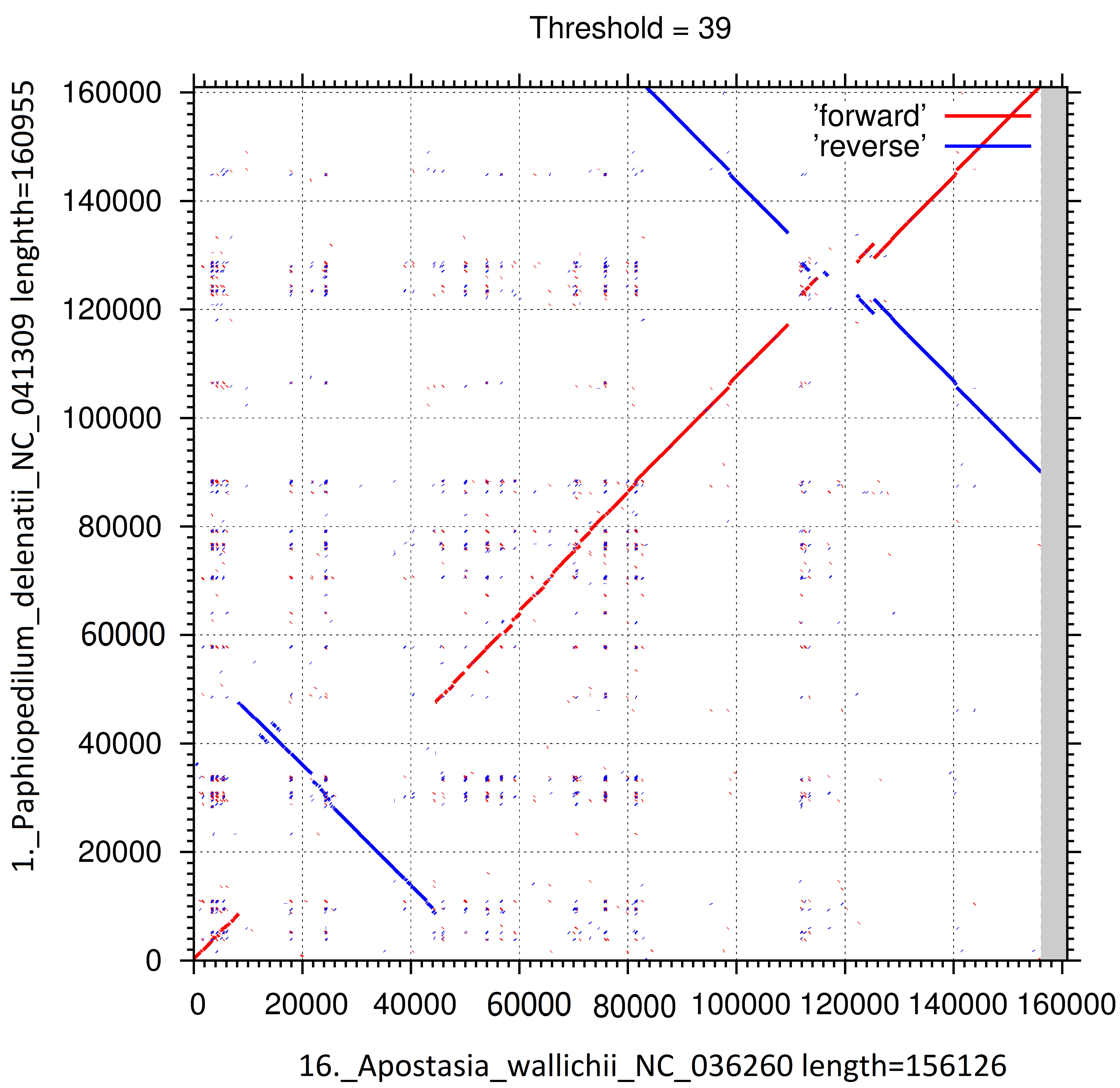

Threshold = 39
'forward'
'reverse'
1._Paphiopedilum_delenatii_NC_041309 length=160955
16._Apostasia_wallichii_NC_036260 length=156126
0
20000
40000
60000
80000
100000
120000
140000
160000

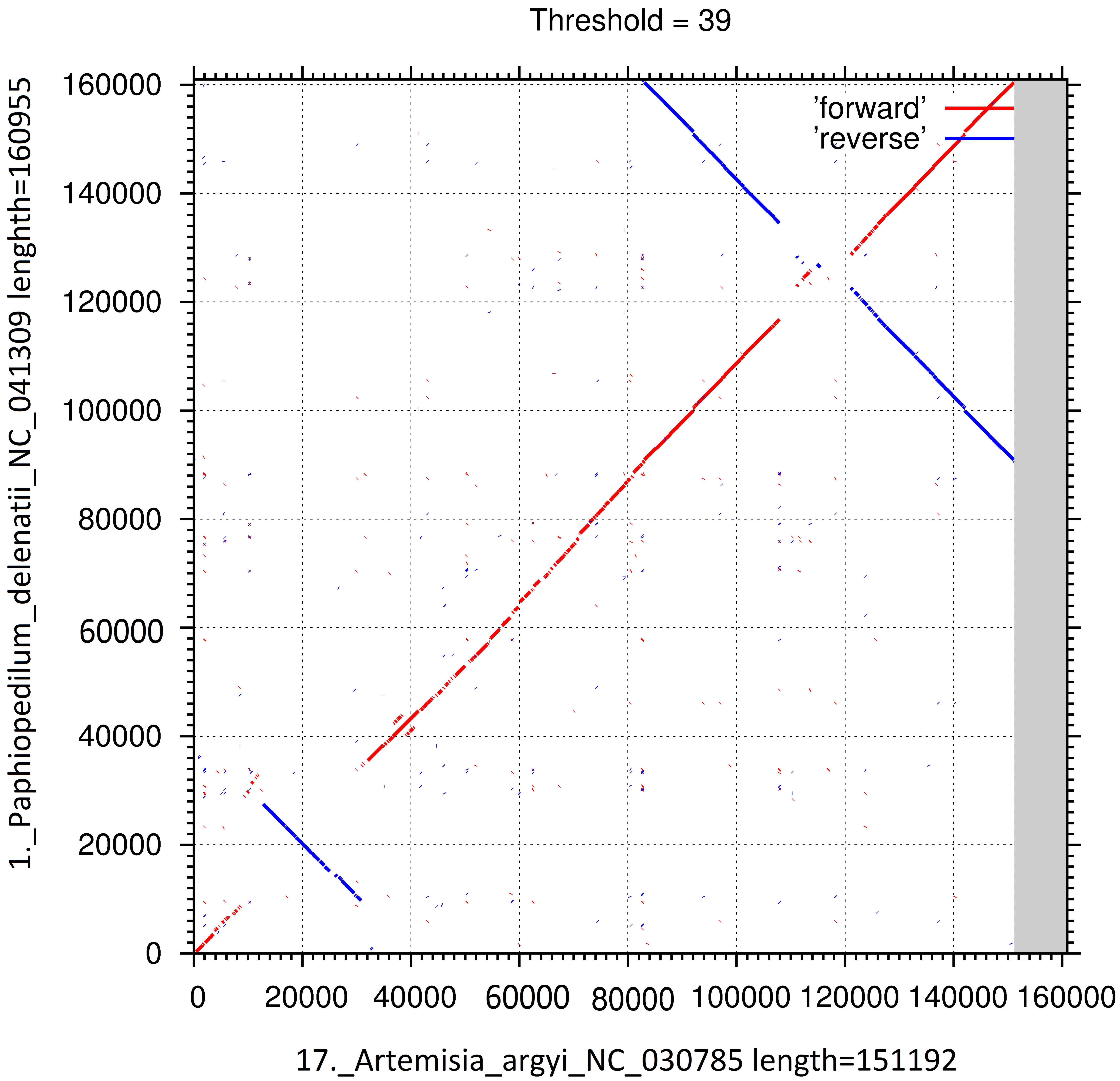

Threshold = 39
'forward'
'reverse'
1._Paphiopedilum_delenatii_NC_041309 length=160955
17._Artemisia_argyi_NC_030785 length=151192
0
20000
40000
60000
80000
100000
120000
140000
160000

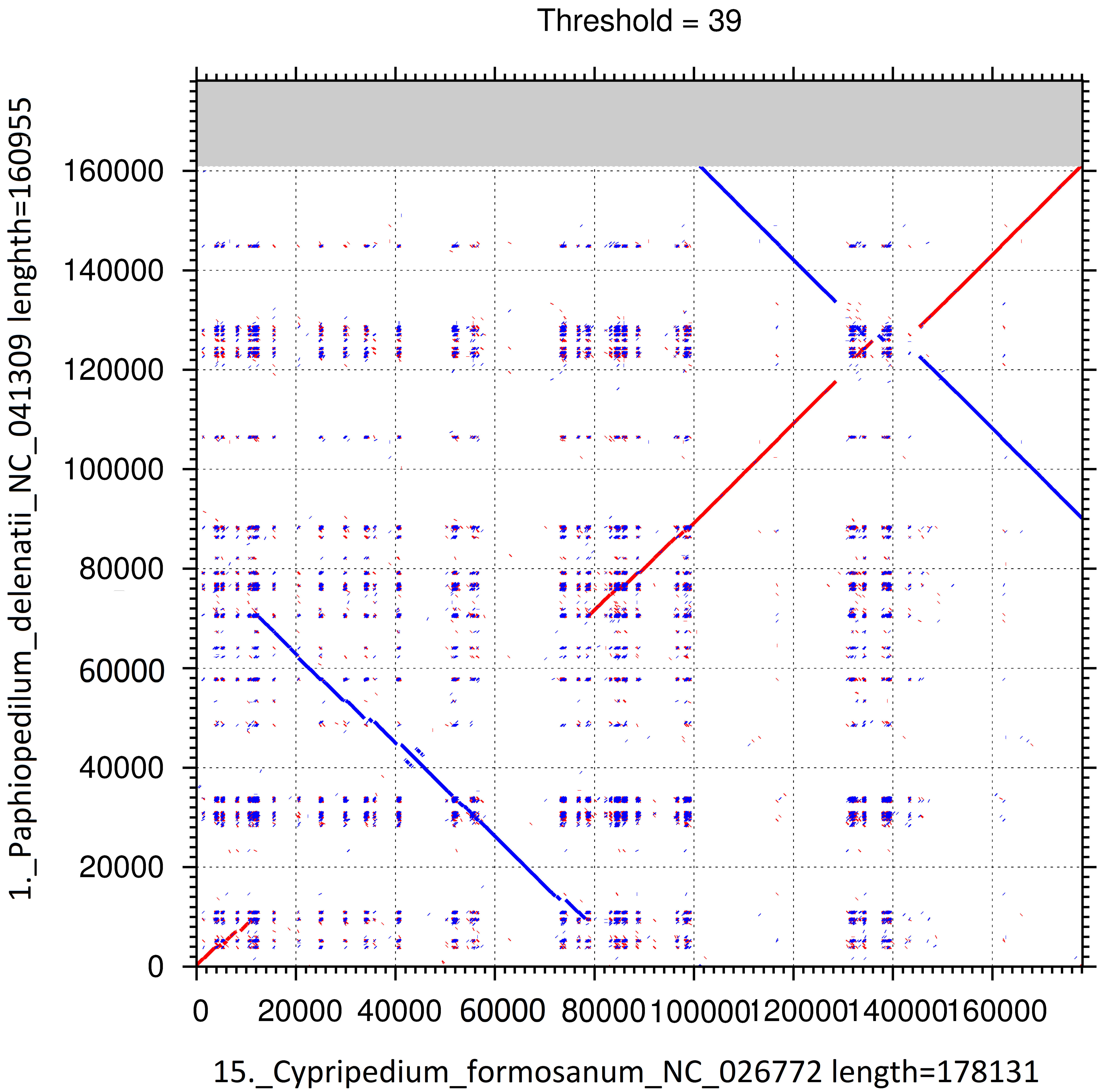
Threshold = 39
1._Paphiopedilum_delenatii_NC_041309 lenghth=160955
15._Cypripedium_formosanum_NC_026772 length=178131
0
20000
40000
60000
80000
100000
120000
140000
160000
0
20000
40000
60000
80000
100000
120000
140000
160000

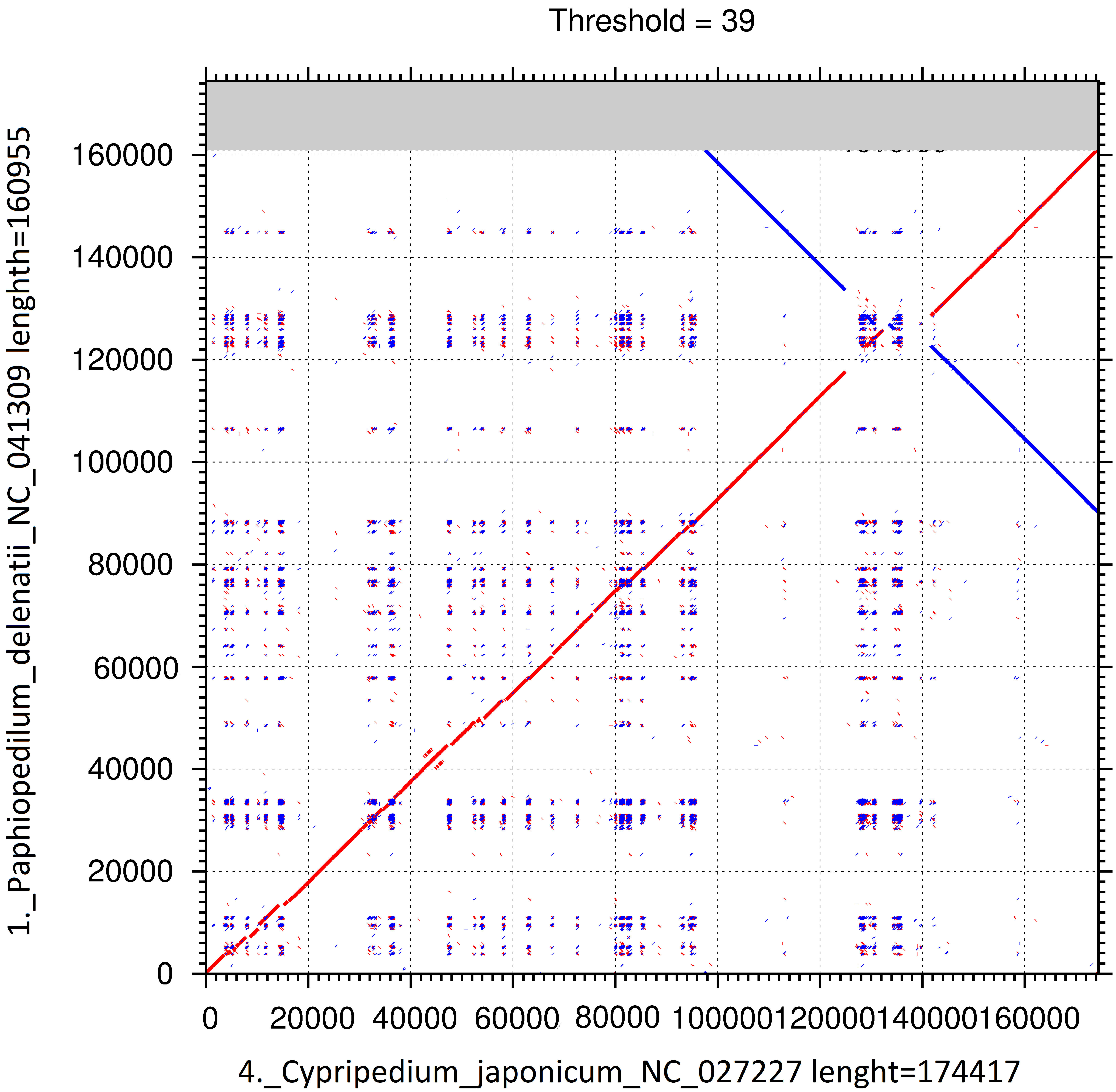
Threshold = 39
1._Paphiopedilum_delenatii_NC_041309 lenghth=160955
4._Cypripedium_japonicum_NC_027227 lenght=174417
0
20000
40000
60000
80000
100000
120000
140000
160000

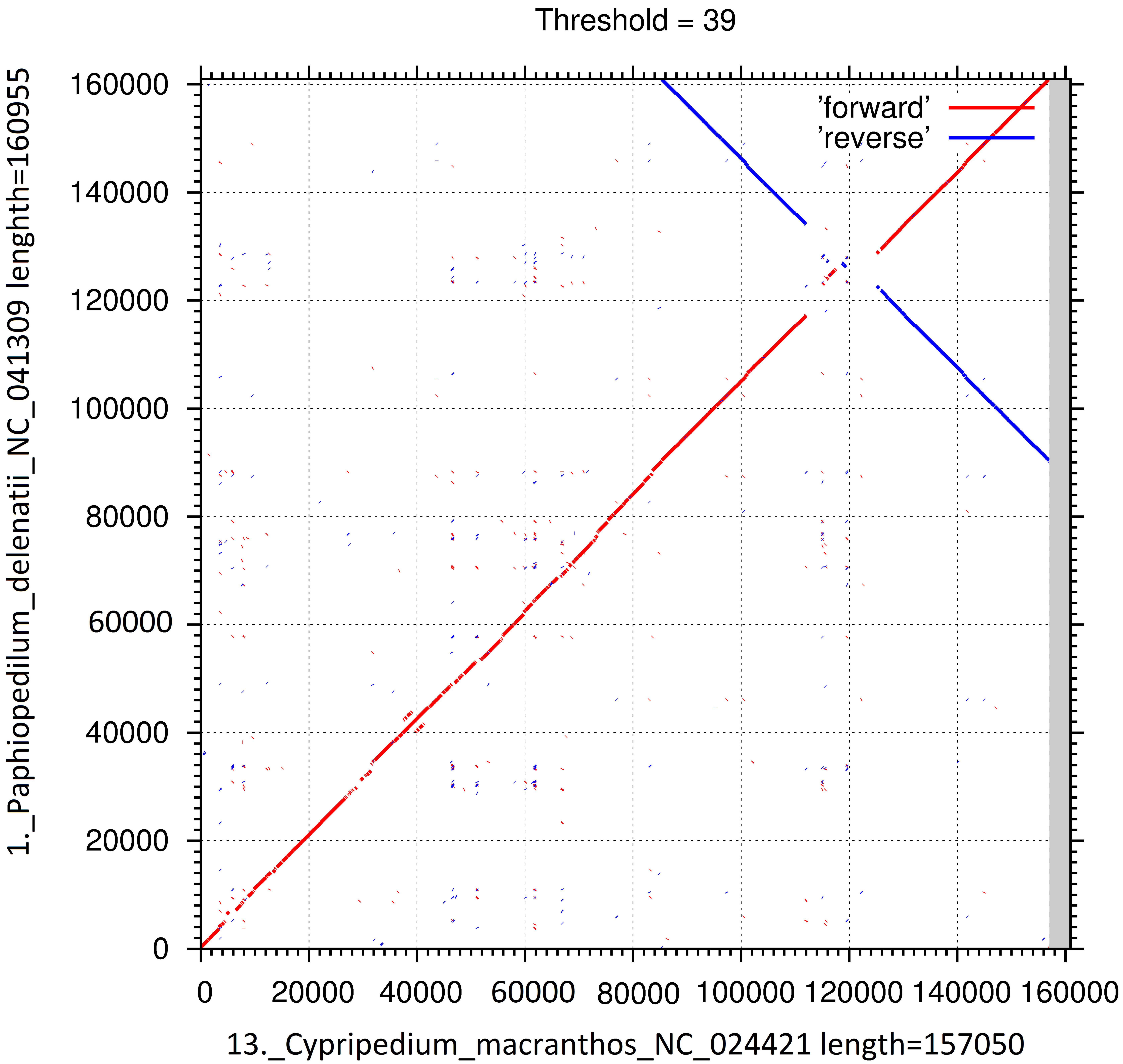

Threshold = 39
'forward'
'reverse'
1._Paphiopedilum_delenatii_NC_041309 length=160955
13._Cypripedium_macranthos_NC_024421 length=157050
0
20000
40000
60000
80000
100000
120000
140000
160000
