# Supplementary material for: Complete Chloroplast Genome of Paphiopedilum delenatii and Phylogenetic Relationships among Orchidaceae
Source: Plants (Basel). 2020 Jan 2;9(1):61. doi: 10.3390/plants9010061 (PMC7020410; doi:10.3390/plants9010061)
Supplement: Supplementary file 1 [file Supplementary Figure S4.pdf › Supplementary Figure S4.pdf]

Supplementary Figure S4. Amino acid polymorphism and number of variable sites between *C. macranthos* and other Orchidaceae species using *rbc*L sequence

Amino acid polymorphism	9	21	33	68	78	89	95	97	116	142	143	219	222	224	225	226	230	245	251	265	326	328	340	341	353	359	363	Number of amino acid variation	Number of nucleotide variation
Cypripedium_macranthos_NC_024421	A	R	D	T	D	P	T	F	M	P	A	V	A	A	I	Y	A	A	I	V	I	A	E	M	F	S	F		
Cypripedium_japonicum_NC_027227	.	K	.	.	.	A	N	Y	L	.	S	L	T	.	L	.	T	G	.	.	.	S	.	.	.	.	.	12	59
Cypripedium_formosanum_NC_026772	.	K	.	.	.	A	N	Y	L	.	S	L	T	.	L	.	T	G	.	.	.	S	.	.	.	.	.	12	61
Paphiopedilum_delenatii_NC_041309	.	K	.	.	.	A	N	Y	.	.	S	L	.	.	L	.	T	G	.	.	.	S	.	.	.	.	.	10	61
Paphiopedilum_armeniacum_NC_026779	.	K	.	.	.	A	N	Y	.	.	S	L	.	.	L	.	T	G	.	.	.	S	.	.	.	.	.	10	63
Paphiopedilum_dianthum_NC_036958	.	K	.	.	.	A	N	Y	.	.	S	L	.	.	L	.	T	G	.	.	.	.	.	.	.	.	.	9	61
Paphiopedilum_niveum_NC_026776	.	K	.	.	.	A	N	Y	.	.	S	L	.	.	L	.	T	G	.	.	.	.	.	.	.	.	.	9	61
Phragmipedium_longifolium_NC_028149	T	K	.	.	.	A	N	Y	.	.	S	L	.	.	.	.	.	G	.	.	.	.	.	.	.	.	.	8	54
Apostasia_wallichii_NC_036260	.	K	.	.	N	A	N	Y	.	.	S	.	.	.	L	.	.	G	.	.	.	.	D	.	.	.	.	9	64
Vanilla_pompona_NC_036809	.	K	.	.	.	.	N	Y	.	T	S	.	.	.	.	.	.	G	.	.	.	.	.	L	.	.	.	7	68
Anoectochilus_emeiensis_NC_033895	.	K	S	A	.	.	N	Y	.	T	S	L	.	S	L	.	.	G	M	.	V	G	D	.	Y	N	Y	18	73
Platanthera_japonica_NC_037440	.	K	.	.	.	A	N	Y	.	.	.	L	.	S	.	.	.	G	.	.	.	.	.	.	.	.	.	7	61
Eulophia_zollingeri_NC_037212	.	K	.	.	.	A	N	Y	.	T	S	L	.	.	L	.	.	G	M	.	.	.	.	.	.	.	.	10	72
Calanthe_davidii_NC_037438	.	K	.	.	.	V	N	Y	.	T	S	L	.	.	L	.	.	G	M	.	.	.	.	.	.	.	.	10	63
Cattleya_crispata_NC_026568	.	K	.	.	.	V	N	Y	.	T	S	L	.	.	L	F	.	G	M	I	.	.	.	.	.	.	.	12	69
Neottia_ovata_NC_030712	.	K	.	.	.	A	N	Y	.	T	S	L	.	.	L	.	.	G	M	.	.	.	.	.	.	.	.	10	65
